# Metabolic dysregulation in pulmonary fibrosis: insights into amino acid contributions and therapeutic potential

**DOI:** 10.1038/s41420-025-02715-2

**Published:** 2025-08-27

**Authors:** Hongyu Zheng, Lei Zhang, Congjian Wang, Yi Wang, Chenxi Zeng

**Affiliations:** 1 Department of Pulmonary and Critical Care Medicine, NHC Key Laboratory of Respiratory Diseases, Tongji Medical College and State Key Laboratory for Diagnosis and Treatment of Severe Zoonotic Infectious Diseases, Tongji Hospital, Tongji Medical College, Huazhong University of Science and Technology, Wuhan, China; 2 Department of Thoracic Surgery, Tongji Hospital, Tongji Medical College, Huazhong University of Science and Technology, Wuhan, China

**Keywords:** Respiratory tract diseases, Metabolic disorders

## Abstract

Idiopathic pulmonary fibrosis (IPF) is a progressive and life-threatening interstitial lung disease characterized by excessive extracellular matrix deposition and fibroblast activation. Emerging evidence suggests that amino acid metabolism plays a crucial role in the pathogenesis of pulmonary fibrosis. Key amino acids, including arginine, proline, and glutamine, contribute to the regulation of fibroblast activity and collagen synthesis, all of which are essential for fibrotic progression. Studies in experimental models of pulmonary fibrosis have demonstrated significant metabolic dysregulation, further highlighting its relevance in disease development. Moreover, targeting amino acid metabolism has emerged as a promising therapeutic strategy, with novel drugs and interventions designed to modulate metabolic pathways showing potential in preclinical and clinical studies. This review explores the intricate interplay between amino acid metabolism and pulmonary fibrosis, discusses its implications for disease progression, and evaluates the therapeutic prospects of metabolic interventions in IPF management. Understanding these metabolic mechanisms may pave the way for more effective and personalized treatment strategies for IPF.

## Facts

- Amino acid metabolism is dysregulated in IPF.
- Key amino acids contribute to fibrotic progression.
- Targeting amino acid metabolism shows therapeutic potential.

## Open Questions

- How do specific amino acid metabolic pathways interact with known fibrotic signaling cascades?
- Can metabolic profiling be used to stratify IPF patients for personalized therapy?
- What are the long-term effects and safety profiles of metabolic interventions targeting amino acids in IPF patients?

## Introduction

Idiopathic pulmonary fibrosis (IPF) is a chronic, progressive interstitial lung disease that significantly impacts patients’ life quality and imposes a substantial economic burden. Dyspnea is the most common presenting symptom, often prompting initial medical consultation, while chronic dry cough and velcro-like crackles on auscultation are also characteristic clinical features [1, 2]. The reported general prevalence and incidence range from 0.5 to 27.9/100,000 and 0.2 to 8.8/100,000, respectively, and the disease’s prevalence seems to be rising, particularly in people over 65 [2, 3]. Compared to South America and East Asia, the incidence of IPF is more common in North America and Europe [1, 2]. IPF has a poor prognosis with a median survival time of 3–5 years due to the lack of viable treatment alternatives [4–6].

Amino acid metabolism is a fundamental biological process that not only provides building blocks for protein synthesis, but also supports various cellular functions, including redox homeostasis, energy production, and epigenetic regulation [7–9]. Amino acids can be obtained through dietary intake, tissue protein breakdown, and endogenous biosynthesis [10]. Beyond their role in general metabolism, emerging evidence suggests that amino acid metabolic reprogramming plays a crucial role in the pathogenesis of IPF.

IPF is characterized by excessive extracellular matrix (ECM) deposition—mainly collagen—which leads to irreversible scarring of the lung and progressive respiratory failure [11, 12]. Several amino acids are directly involved in collagen biosynthesis or regulate fibrogenic pathways. For instance, L-arginine is metabolized by arginase to produce L-ornithine, a precursor for both polyamines and L-proline—two metabolite groups intimately associated with fibrosis. Proline and its hydroxylated form, hydroxyproline, constitute approximately one-third of collagen, making proline availability a limiting factor for collagen synthesis and ECM accumulation [13–16]. In parallel, glycine, another major component of collagen, is synthesized from serine, which is derived from the glycolytic intermediate 3-phosphoglycerate via the enzymes phosphoglycerate dehydrogenase (PHGDH), phosphoserine aminotransferase 1 (PSAT1), and phosphoserine phosphatase (PSPH), and subsequently converted by serine hydroxymethyltransferase 1/2 (SHMT1/2) [17, 18]. These pathways not only fuel collagen production but also intersect with one-carbon metabolism, supporting nucleotide biosynthesis and methylation reactions. Moreover, amino acid metabolism regulates the redox balance through the generation of nicotinamide adenine dinucleotide phosphate (NADPH) and reduced glutathione (GSH), which are essential for cellular defense against oxidative stress—a key driver in IPF progression [7, 19]. Given their dual role in both structure and signaling, amino acids are increasingly recognized as central players in fibrogenesis. In this review, we highlight the emerging evidence linking amino acid metabolic pathways—particularly those involving arginine, proline, glycine, and serine—to the initiation and progression of pulmonary fibrosis. A deeper understanding of these pathways may uncover novel therapeutic targets for IPF, especially those aimed at reversing or modulating metabolic dysregulation in fibrotic lungs.

## Pathogenesis of IPF

### Genetic and Environmental Factors

IPF is driven by a combination of genetic susceptibility and environmental exposures. Key susceptibility genes are involved in pathways such as mucin production, surfactant metabolism, telomere maintenance, immune regulation, and epithelial integrity [20]. Among these, the MUC5B promoter variant (rs35705950) is the most prominent, conferring a greater than threefold increased risk for IPF and exceeding the effect of all other known variants combined [21, 22]. Other associated genes include TOLLIP, DSP, and AKAP13, which influence immune responses, epithelial stability, and tissue remodeling [23–25].

On the environmental side, viral and bacterial infections (e.g., EBV, CMV, and Streptococcus) have been implicated in chronic alveolar injury, a key initiating factor in fibrosis [26, 27]. Additionally, exposures such as cigarette smoke, agricultural chemicals, organic and inorganic dusts, and air pollution are associated with increased IPF risk, likely by exacerbating epithelial damage and promoting aberrant repair [20, 28]. These genetic and environmental factors interact to trigger persistent epithelial injury and dysregulated repair, central to the fibrotic remodeling seen in IPF.

### “Injure-Repair” process

Fibrosis is characterized by excessive deposition of ECM components and represents a maladaptive response to chronic or repeated tissue injury [29, 30]. Katzenstein et al. first proposed that pulmonary fibrosis results from failed repair following epithelial injury, a concept now widely supported [31, 32]. In IPF, environmental factors such as cigarette smoke, air pollution, and microbial infections damage alveolar epithelial cells (AECs) and microvascular endothelial cells, initiating an aberrant “injury–repair” cascade [33–35]. Damaged AECs release pro-inflammatory and pro-fibrotic mediators, including interleukin (IL) -1β and tumor necrosis factor (TNF) -ɑ, which promote fibroblast recruitment, inflammatory cell activation (e.g., neutrophils, Th17, gdT cells), and myofibroblast differentiation, ultimately leading to ECM accumulation and fibrotic tissue remodeling [20, 29, 33, 34]. Myofibroblasts, the key effector cells in fibrosis, arise from fibroblasts, pericytes, endothelial cells, or epithelial cells via epithelial–mesenchymal transition (EMT) under stimulation by transforming growth factor (TGF) -β, clotting factors, and mechanical stress [11, 29, 34, 36]. They secrete large amounts of collagen and fibronectin, disrupt the basement membrane, and induce epithelial apoptosis, resulting in irreversible architectural distortion. As fibrosis progresses, tissue stiffening and local hypoxia further promote persistent epithelial injury and myofibroblast activation, creating a vicious cycle that drives disease progression [37].

### TGF-β signaling

Fibrosis is driven by a complex interplay of pathological processes, including activation of TGF-β, dysregulated chemokine expression, metabolic dysfunction, cellular senescence, mitochondrial and endoplasmic reticulum (ER) stress, autophagy, DNA damage, and apoptosis [20, 33, 36, 37]. Among these, TGF-β signaling is widely recognized as a central mediator of fibrotic progression and has emerged as a key therapeutic target [33, 36]. TGF-β is secreted by AECs, endothelial cells, fibroblasts, myofibroblasts, and various immune cells, and it promotes EMT, metabolic reprogramming, and excessive ECM production—hallmark features of fibrosis [36, 37].

TGF-β is activated through interactions with glycoprotein A repetitions predominant (GARP), integrins, proteases, and other TGF-β-binding proteins, enabling its subsequent binding to the TGF-β receptor family (TβR) [36]. The TβR complex consists of three isoforms: TβR I and TβR II, which are serine/threonine and tyrosine kinases, and TβR III, which lacks intrinsic kinase activity and functions as a co-receptor to facilitate ligand presentation [38]. Upon ligand binding, TβR II phosphorylates and activates TβR I, forming a heteromeric receptor complex that initiates downstream signaling through both SMAD-dependent and SMAD-independent pathways, such as ERK, p38, JNK, and PI3K-Akt cascades [33, 36, 39].

In the canonical SMAD pathway, activated TβR I phosphorylates receptor-regulated SMADs (R-SMADs), primarily SMAD2 and SMAD3. These phosphorylated R-SMADs dissociate from the receptor and form a trimeric complex with SMAD4, which translocates into the nucleus to regulate transcription of fibrosis-related genes, including α-smooth muscle actin (α-SMA) and collagen I [40, 41]. SMAD7 functions as an inhibitory SMAD that competes with R-SMADs for receptor binding, thereby negatively regulating the pathway [40]. Interestingly, inhibition of adrenoceptor beta 2 (ADRB2) has been shown to attenuate fibrosis by suppressing SMAD2/3 phosphorylation and promoting proteasomal degradation of phospho-SMAD2/3 in lung fibroblasts [42].

**Fig. 1:**
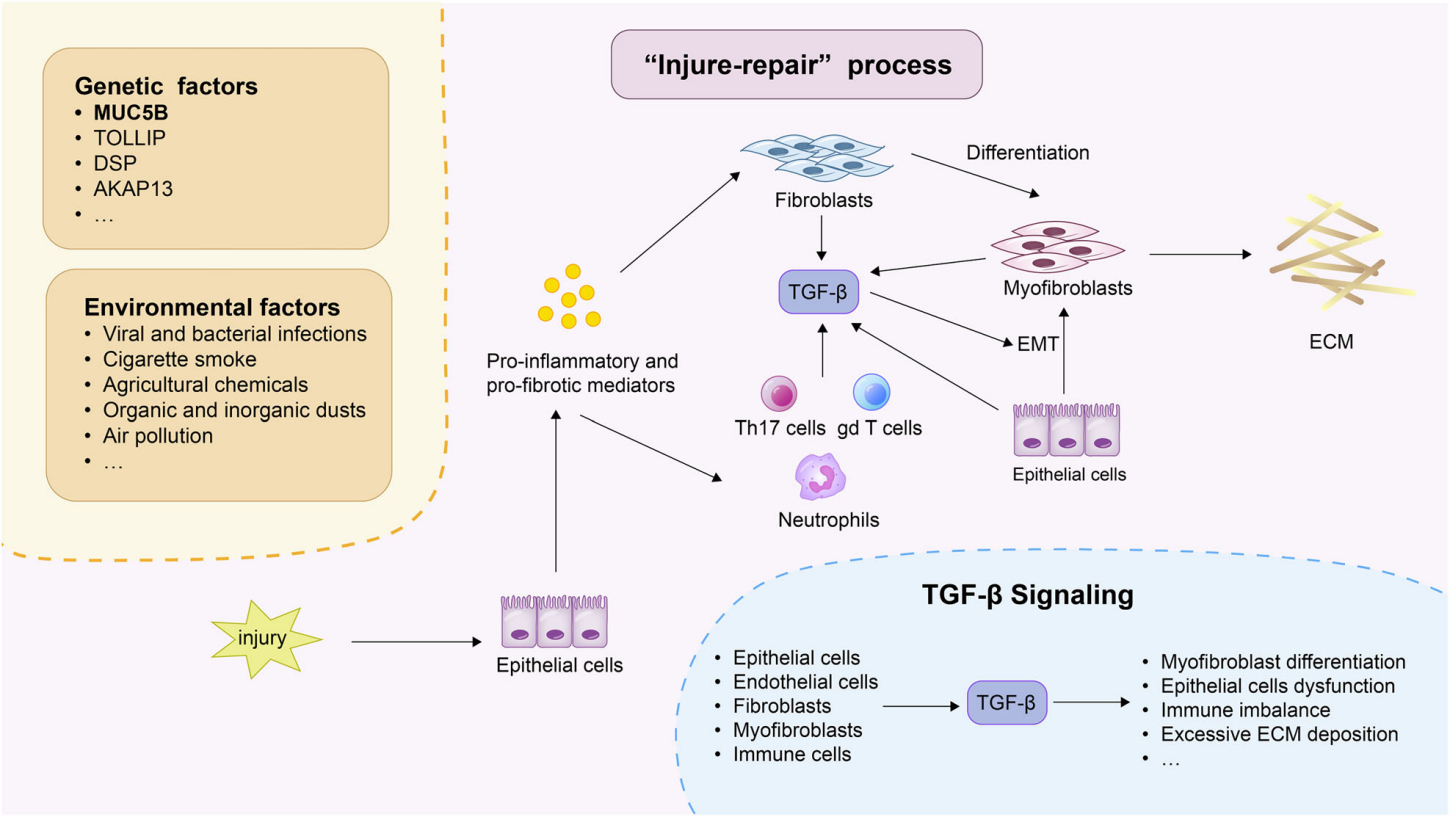
Pathogenesis of pulmonary fibrosis. Irritants damage lung epithelial cells, prompting them to release pro-inflammatory and pro-fibrotic cytokines, which leads to the activation of immune cells. These immune cells, in turn, release additional cytokines, resulting in the differentiation of fibroblasts into myofibroblasts and the induction of EMT. Myofibroblasts synthesize ECM components, ultimately contributing to the development of pulmonary fibrosis.

Beyond the canonical SMAD pathway, TGF-β also signals through non-SMAD mechanisms. The PI3K/AKT axis drives fibrotic responses via AKT/mTOR and PAK2/c-Abl signaling [43, 44], while the MEK/ERK pathway contributes to pulmonary fibrosis, particularly under Bmi-1 deficiency [45]. Recent studies have also identified NP-011, a truncated variant of milk fat globule-EGF factor 8, which significantly reduces TGF-β1-induced fibrogenesis and collagen accumulation by specifically inhibiting ERK signaling [46]. Together, these interconnected signaling cascades reshape transcriptional programs in fibroblasts, AECs, and endothelial cells, promoting myofibroblast differentiation, epithelial cell dysfunction, immune dysregulation, and excessive ECM deposition, which collectively drive the onset and progression of pulmonary fibrosis [33, 39] (Fig. 1).

## Amino acids metabolism

### Amino acid metabolism in cellular functions

Amino acids, traditionally recognized as the fundamental components of protein biosynthesis, also play versatile roles as precursors for enzymes, signaling molecules, and essential metabolic intermediates that support cellular homeostasis and tissue remodeling [8, 47, 48]. Beyond their structural functions, amino acids are intricately involved in energy production, redox regulation, and signaling cascades—processes that are critically implicated in the onset and progression of fibrosis. In the fibrotic context, metabolic pathways driven by specific amino acids directly support cellular proliferation, differentiation, and excessive ECM deposition, which are hallmark features of fibrogenesis.

Among these, glutamine and arginine have emerged as central players in fibroblast activation and metabolic reprogramming. Glutamine, through conversion to glutamate and subsequently to α-ketoglutarate (α-KG), not only fuels the tricarboxylic acid (TCA) cycle for energy generation but also contributes carbon skeletons for lipid, carbohydrate, and nucleotide biosynthesis, thus sustaining the high metabolic demands of activated fibroblasts [8]. Additionally, folate-mediated one-carbon metabolism, closely linked to glutamine availability, is essential for nucleotide synthesis and redox balance via NADPH production [7, 49]. Polyglutamylation of folate in cells ensures its metabolic retention and facilitates enzyme specificity [49], thereby supporting mitochondrial function and oxidative stress tolerance—both crucial for the persistence and activation of fibrogenic cells [7].

Arginine metabolism is equally pivotal in the fibrotic process. Through its conversion to ornithine and proline, arginine directly supplies the substrates required for collagen biosynthesis, positioning it as a metabolic driver of ECM accumulation [13–16]. This pathway is tightly regulated by enzymes such as ornithine aminotransferase (OAT) and pyrroline-5-carboxylate reductase (PYCR), which control proline production and, consequently, the rate of collagen deposition [13–16]. Beyond its metabolic role, arginine also influences immune regulation within the fibrotic microenvironment. It modulates TGF-β signaling, a key pro-fibrotic pathway [50], and regulates the functional capacity of T cells by maintaining TCR CD3-ζ chain expression and cyclin D3/cdk4-mediated proliferation [51, 52]. These findings suggest that arginine availability is not only a metabolic substrate but also a determinant of immune competence and myofibroblast activation.

Collectively, the metabolic flexibility provided by glutamine and arginine supports both the bioenergetic and biosynthetic needs of fibrotic tissues while sustaining a permissive microenvironment for chronic immune dysregulation and fibroblast persistence. Targeting these amino acid-driven metabolic circuits may offer novel therapeutic opportunities in fibrotic diseases such as IPF, where metabolic vulnerabilities can be exploited to disrupt fibroblast activation and excessive ECM remodeling.

### Amino acids support lung repair and oxidative homeostasis

Amino acids are fundamental for protein synthesis in lung cells, supporting tissue integrity and facilitating repair after injury. Beyond their structural role, they contribute to energy production by entering the TCA cycle through deamination [8]. Amino acids also participate in the synthesis of pulmonary surfactants, which are essential for reducing alveolar surface tension and maintaining normal lung compliance [53, 54]. Pulmonary surfactant, composed of phospholipids, triglycerides, cholesterol, free fatty acids, and four key proteins (SP-A, SP-B, SP-C, and SP-D), prevents alveolar collapse and supports effective gas exchange [54–56]. Amino acids are critical for the biosynthesis of these surfactant proteins, which interact with phospholipids to regulate surfactant stability and surface activity [56].

In addition to their structural and metabolic roles, amino acids are closely involved in oxidative stress regulation. Glutamate, cysteine, and glycine serve as precursors for GSH and NADPH, key molecules that neutralize reactive oxygen species (ROS) and maintain cellular redox balance [9, 57]. While moderate ROS levels are necessary for physiological signaling and tissue remodeling, excessive oxidative stress can disrupt redox homeostasis, leading to persistent inflammation and fibrosis in the lung [58–60]. Amino acid supplementation has shown protective potential in this context. For example, glutamine can boost GSH production through activation of Nrf2 pathways, attenuating oxidative lung injury [61]. Arginine has been reported to alleviate pulmonary inflammation by improving mitochondrial function, enhancing ATP generation, and reducing DNA damage [62, 63]. Additionally, glycine can inhibit NLRP3 inflammasome activation, thereby reducing inflammatory lung injury [64], and glutamine supports tissue regeneration by promoting immune-driven growth factor release [65].

Collectively, these findings suggest that amino acid metabolism plays a multifaceted role in lung health, contributing not only to structural maintenance and energy balance but also to redox regulation, immune modulation, and tissue repair. Targeting amino acid pathways may represent a promising therapeutic approach to mitigate oxidative stress, control inflammation, and promote recovery in lung injury and fibrosis.

## Amino acid metabolism and pulmonary fibrosis

### Arginine plays a key role in pulmonary fibrosis

#### Arginine–proline metabolism in pulmonary fibrosis

Emerging evidence indicates that the arginine–proline metabolic axis is notably upregulated in pulmonary fibrosis, contributing to excessive ECM deposition and disease progression [66–70]. Given that proline and hydroxyproline together account for approximately one-third of collagen’s amino acid content, increased proline biosynthesis is now considered a key metabolic driver of fibrotic remodeling [13–16]. In fibrotic lungs, arginine is first converted to ornithine via arginase, and ornithine is subsequently transformed into proline through the sequential actions of OAT and PYCR [13, 14]. Both OAT and PYCR1 are markedly elevated in IPF lung tissues and fibroblasts, with their expression levels correlating with deteriorating lung function and poor prognosis [71, 72].

Mechanistically, OAT not only promotes TGF-β1 activation but also enhances Smad and non-Smad signaling, supporting sustained fibrotic responses. In addition, OAT induces proline dehydrogenase (PRODH), leading to mitochondrial ROS production, which further amplifies TGF-β1 signaling and tissue fibrosis [71]. PYCR1, frequently found in complex with kindlin-2, also plays a critical role in mitochondrial proline biosynthesis and fibroblast activation. Kindlin-2 supports collagen matrix assembly by stabilizing the PYCR1 complex. Both genetic silencing of kindlin-2 and pirfenidone treatment have been shown to reduce PYCR1 activity, proline production, and fibrosis severity in vivo [14, 73].

Arginine transport is another essential checkpoint in this metabolic circuit. The cationic amino acid transporter-2 (CAT-2) mediates arginine uptake, which is required for arginase activity and subsequent collagen production, particularly in macrophages [74]. Arginase-1, predominantly expressed in macrophages, and arginase-2, localized in various cells including myofibroblasts, both contribute to proline generation. Notably, arginase-2 colocalizes with heat shock protein 47 (Hsp47), a collagen-specific chaperone involved in collagen maturation [15]. Pharmacological inhibition of arginase suppresses TGF-β1-induced collagen synthesis in both murine and human fibrotic models [75, 76]. Additionally, a positive feedback loop involving IL-6 secretion from fibroblasts and arginase-1 induction in macrophages further amplifies the fibrotic cascade [76].

Beyond its role in proline metabolism, arginine also gives rise to polyamines such as spermidine and creatine, which exhibit anti-fibrotic properties. Interestingly, the levels of these polyamines are significantly reduced in fibrotic lungs, and their supplementation has been shown to alleviate oxidative stress and attenuate fibrosis [16, 77–79]. Another arginine-related pathway involves carbamoyl phosphate synthetase 1 (CPS1), a key enzyme in the urea cycle that regenerates arginine through citrulline recycling. CPS1 expression is notably increased in arsenite-induced lung fibrosis and contributes to enhanced proline metabolism. Inhibition of CPS1 or reduction of ammonia levels effectively suppresses collagen production and mitigates fibrosis in experimental models [80]. The arginine–proline metabolic network plays a central role in sustaining fibroblast activation, ECM overproduction, and oxidative stress in pulmonary fibrosis. Targeting this pathway at multiple nodes—including arginine transport, proline synthesis, and polyamine metabolism—may offer promising therapeutic strategies to halt fibrotic progression.

#### Arginine–NOS pathway in the pathogenesis of pulmonary fibrosis

L-arginine is a key metabolic node involved in multiple pathways that influence the progression of pulmonary fibrosis. Arginase converts L-arginine into ornithine and proline, fueling collagen synthesis and ECM accumulation [13]. And nitric oxide synthases (NOS) metabolize L-arginine to nitric oxide (NO), a molecule with dual roles in fibrosis. Elevated NOS2 expression in IPF increases NO production, which can exacerbate fibrosis by activating inflammatory pathways such as NF-κB and NLRP3 [13, 81]. However, NO is not universally detrimental. Studies have shown that NOS knockout aggravates bleomycin (BLM)-induced fibrosis, whereas NO supplementation mitigates tissue damage [82]. This paradox may be partially explained by the cellular source: endothelial NOS (eNOS)-derived NO appears protective, while inducible NOS (iNOS) from macrophages tends to promote fibrosis [83].

The enzyme dimethylarginine dimethylaminohydrolase (DDAH) further regulates NO production by degrading asymmetric dimethylarginine (ADMA), an endogenous NOS inhibitor. DDAH is upregulated in IPF, leading to decreased ADMA levels and increased NOS2 activity, thereby enhancing NO production and contributing to fibrotic progression [81]. Interestingly, inhibiting DDAH reduces TGF-β/Smad-mediated collagen synthesis in fibroblasts and limits alveolar type II (ATII) cell proliferation in an ADMA-dependent manner [81, 84].

Arginine supplementation has demonstrated protective effects in BLM-induced lung fibrosis, improving antioxidant capacity, dampening inflammation, and restoring mitochondrial function. It also modulates the HO-1/PPARγ/Wnt/β-catenin signaling axis, promoting NO production and E-cadherin expression to suppress fibrotic responses [85]. Combined therapy with L-arginine and L-norvaline further enhances these effects by reducing IL-17A expression, lowering peroxynitrite-induced oxidative stress, and inhibiting iNOS activity to restore arginine metabolic balance [86].

In addition to its direct metabolism, arginine can be regenerated via the urea cycle through argininosuccinate synthase 1 (ASS1) and argininosuccinate lyase (ASL). Notably, citrulline supplementation restores fibroblast activation under arginine-deprived conditions, whereas ornithine and glutamine provide essential precursors for polyamine biosynthesis in fibroblasts [50]. However, ASS1 is frequently downregulated in fibrotic lung tissues, which promotes fibroblast activation through STAT3 signaling and drives an aggressive, apoptosis-resistant phenotype [87, 88]. Importantly, arginine deprivation therapy has been shown to alleviate fibrosis in vivo and enhance the antifibrotic efficacy of nintedanib [88].

Overall, the role of arginine metabolism in pulmonary fibrosis is highly context-dependent, influenced by the interplay between the arginase and NOS pathways and the regulatory control of enzymes such as DDAH and ASS1. Carefully targeting specific metabolic branches within the arginine network may offer promising therapeutic avenues for IPF, allowing modulation of fibrosis while preserving essential immune and vascular functions.

#### Arginine-modifying enzymes in pulmonary fibrosis

Beyond its metabolic roles, arginine is subject to post-translational modifications, such as methylation and citrullination, which significantly impact pulmonary fibrosis. Several studies have identified protein arginine methyltransferase (PRMT) 1, PRMT4, PRMT5, and PRMT7 as key regulators of fibroblast activation and differentiation in fibrosis. Inhibiting PRMT1/4 suppressed TGF-β-induced myofibroblast activation and fibrinogen fiber formation, without affecting normal fibroblasts [89]. PRMT1 expression was elevated in fibroblasts from IPF patients and BLM-treated mice, but not in total lung tissue, suggesting cell-type specificity [90]. IL-4, a pro-fibrotic cytokine, upregulated PRMT1 and enhanced fibroblast migration. Notably, depletion (rather than pharmacologic inhibition) of PRMT1 significantly suppressed fibroblast proliferation, linking intracellular arginine methylation to fibrotic remodeling [90]. PRMTs also regulate immune and epithelial components of fibrosis. For example, PRMT1-mediated STAT6 methylation, induced by hypoxia-inducible factor (HIF)-1α, promoted M2 macrophage polarization, contributing to fibrotic signaling in lung epithelial cells [91]. In alveolar myofibroblasts, PRMT7 was shown to monomethylate histone H4 (H4R3me1) at the Forkhead box M1 (Foxm1) promoter, activating its transcription and promoting myofibroblast proliferation and elastin deposition. PRMT7-deficient mice exhibited impaired myofibroblast differentiation and ECM remodeling [92].

Additionally, in IPF lungs, mesenchymal progenitor cells (MPCs) display cancer-like features driven by a CD44/Brg1/PRMT5 axis, which epigenetically silences tumor suppressors RBL1 and PTEN, supporting self-renewal and fibrogenesis [93]. Peptidyl arginine deiminase (PAD) enzymes, particularly PAD2 and PAD4, catalyze the citrullination of arginine residues, modulating gene expression and immune responses. In rheumatoid arthritis -associated interstitial lung disease, elevated PAD2 expression in lung fibroblasts promoted a pro-fibrotic phenotype, while overexpression of syndecan-2 (SDC2) inhibited PAD2 and protected against BLM-induced fibrosis [94]. PAD4 was associated with age-related fibrosis in multiple organs, including the lungs, and its inhibition reduced fibrosis by balancing M1/M2 macrophage polarization [95, 96].

**Fig. 2:**
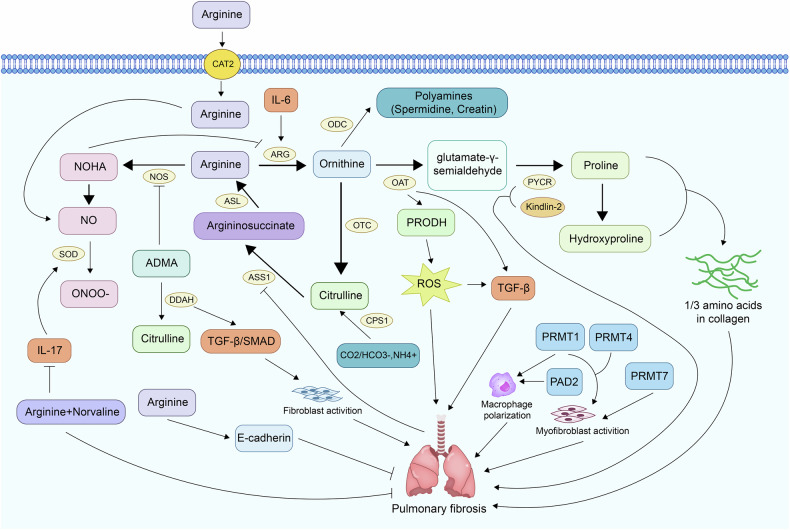
Argine influences pulmonary fibrosis mainly by arginine-proline metabolism, arginine–NOS pathway and post-translational modifications.

These findings highlight the critical roles of arginine-modifying enzymes—notably PRMTs and PADs—in the regulation of fibroblast activity, immune cell polarization, and epigenetic control of gene expression during pulmonary fibrosis. Targeting these enzymes represents a promising strategy for modulating fibrotic remodeling in IPF (Fig. 2).

### Glutamine metabolism and metabolic remodeling in fibroblasts

Glutamine metabolism plays a complex and context-dependent role in the development of IPF. Several studies have shown that TGF-β1 upregulates glutaminase 1 (GLS1), enhancing the conversion of glutamine to glutamate, a critical step for collagen biosynthesis [97–99]. This regulation occurs via both SMAD-dependent and non-canonical (PI3K/mTORC2/PDGFR) signaling pathways, and is reinforced by Sirtuin-7 downregulation, which reduces Forkhead box protein O4 (FOXO4)-mediated suppression of GLS1 expression [100]. In pulmonary fibroblasts, glutamine-derived glutamate contributes to the synthesis of alanine and proline, key substrates for collagen production. Inhibition of GLS1 reduces levels of these amino acids and attenuates fibrotic responses [98, 99]. Glutamate can also be converted to α-KG, which activates mTORC1, promotes proline hydroxylation, and stabilizes collagen in the ECM [97]. However, some studies suggest that α-KG is not strictly essential for collagen production, as alternate pathways involving pyruvate and glutamic-pyruvic transaminase 2 (GPT2) can compensate by regenerating glutamate and alanine, diminishing the antifibrotic effect of GLS1 inhibition [99].

Beyond fibroblasts, glutamine metabolism affects other cell types relevant to fibrosis. In ATII cells, glutamine contributes to ATP production and supports proliferation and differentiation during lung injury repair. Its deficiency leads to impaired regenerative capacity, partly due to reduced α-KG availability [101]. The regulation of GLS1 is not limited to TGF-β. Caveolin-1 (CAV1) suppresses Yes-associated protein 1(YAP1) activity in fibroblasts and thereby downregulates GLS1. Loss of CAV1 in fibrotic lungs enhances GLS1 expression, while treatment with the CAV1-derived peptide CSP7 alleviates fibrosis [102]. Despite the established role of glutamine metabolism in promoting fibrosis, studies have shown context-dependent effects. For instance, glutamine deprivation decreased Col3A1 and PLK1 expression and reduced IPF fibroblast proliferation, migration, and collagen synthesis, effects were partially rescued by α-KG [103]. Epigenetically, α-KG modulates histone H3K27me3 levels, influencing the expression of genes like Col3A1 (but not PLK1), thereby contributing to fibroblast phenotype regulation [103].

Glutamine also impacts fibrosis through anti-apoptotic and stress-response pathways. Its deficiency decreases α-KG, impairing JMJD3/UTX demethylase activity, increasing H3K27me3, and promoting the expression of apoptosis inhibitors such as XIAP and survivin [104]. Additionally, glutamine enhances HSP expression (e.g., HSP70, HSP32), protecting against oxidative and inflammatory damage in the lung [105]. Interestingly, glutamine supplementation can also restore mitochondrial respiration in BLM-injured epithelial cells. However, it simultaneously promotes lactate production, which may activate TGF-β signaling and contribute to fibrogenesis [106]. This dual effect underscores the metabolic plasticity and context-dependent nature of glutamine in IPF.

**Fig. 3:**
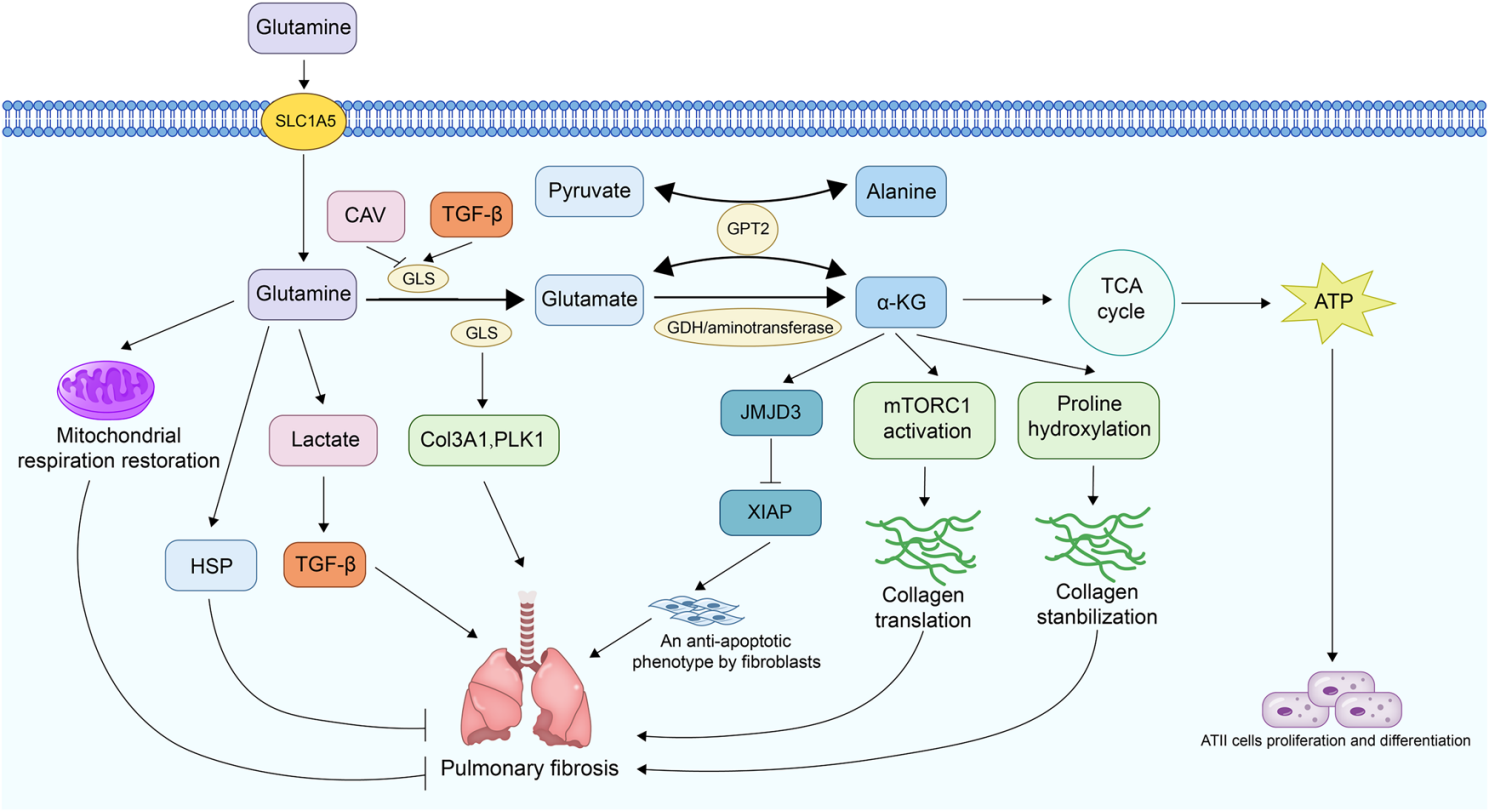
The role of glutamine metabolism and metabolic remodeling in pulmonary fibrosis.

Glutamine metabolism is intricately linked to fibroblast activation, collagen biosynthesis, epigenetic regulation, and cellular stress responses in pulmonary fibrosis. While targeting glutamine pathways—particularly GLS1 and α-KG metabolism—presents promising antifibrotic potential, the cell type-specific and compensatory mechanisms highlight the need for precise therapeutic strategies to effectively harness glutamine-targeted interventions in IPF (Fig. 3).

### Glycine as a collagen precursor in pulmonary fibrosis

Glycine, which constitutes approximately one-third of the amino acids in collagen, plays a pivotal role in ECM accumulation during pulmonary fibrosis [17]. Recent pathway analyses have highlighted that glycine, serine, and threonine metabolism is closely linked to IPF progression [107]. TGF-β signaling is a key driver of this metabolic shift, upregulating enzymes such as PHGDH, PSAT1, PSPH, and SHMT1/2 that are essential for serine–glycine biosynthesis and collagen production [17, 18]. Inhibition of PHGDH effectively reduces glycine availability and has been shown to alleviate fibrosis in experimental models [18].

TGF-β also enhances glycolysis, increasing the generation of 3-phosphoglycerate (3-PG), a critical intermediate that feeds into serine–glycine metabolism [108, 109]. In silica-induced fibrosis models, blocking glycolysis or HIF-1α signaling reduces intracellular glycine levels, which correlates with decreased collagen deposition and attenuated fibrotic responses [110]. These results suggest that metabolic reprogramming in fibrotic fibroblasts is heavily dependent on glucose-derived glycine synthesis. At the transcriptional level, TGF-β activates activating transcription factor 4 (ATF4) through both Smad3 and PI3K–Akt–mTORC1 pathways [108]. ATF4 functions as a central regulator of amino acid metabolism and is essential for the induction of serine–glycine biosynthetic enzymes. mTORC1 further supports glycine synthesis by enhancing glycolytic flux and glucose uptake, contributing to a self-reinforcing cycle that sustains collagen production [108, 109]. Suppressing ATF4 significantly impairs TGF-β-induced glycine and asparagine accumulation, as well as amino acid transport through solute carrier family 7 member 5 (SLC7A5) [111]. Similarly, inhibition of mTOR reduces the levels of glycine, asparagine, glutamine, and proline, all of which are essential for fibroblast activation and matrix deposition [111].

Collectively, these findings position glycine metabolism as a key metabolic hub that integrates glycolysis and collagen biosynthesis in pulmonary fibrosis. The TGF-β–mTORC1–ATF4 axis orchestrates this metabolic shift, supporting fibroblast proliferation, amino acid synthesis, and ECM assembly. Targeting this serine–glycine metabolic pathway or upstream glycolytic regulators may offer a promising therapeutic strategy to disrupt the metabolic support of fibrosis and slow IPF progression.

### Tryptophan metabolites in fibrosis regulation

Tryptophan metabolism plays a complex, context-dependent role in pulmonary fibrosis, influencing ECM deposition, fibroblast activation, and immune responses. Elevated L-tryptophan levels have been observed in fibrotic models and are positively associated with pro-inflammatory cytokines [112]. Tryptophan promotes TGF-β1–induced EMT and fibroblast activation via the mTOR/S6 signaling pathway, exacerbating fibrotic progression [112]. Among tryptophan metabolites, serotonin (5-HT) is known to aggravate fibrosis by enhancing inflammation, neutrophil infiltration, oxidative stress, and upregulating TGF-β1 and type I collagen [113]. In contrast, other tryptophan-derived metabolites exhibit protective effects. Indole-3-acetic acid (IAA), produced by gut microbiota, alleviates fibrosis by suppressing PI3K/AKT/mTOR signaling, enhancing autophagy, and reducing fibroblast activation and epithelial senescence [114]. Similarly, 5-methoxytryptophan (5-MTP) inhibits fibroblast-to-myofibroblast transition and ECM protein expression by downregulating TGF-β/Smad3 and PI3K/AKT pathways, thereby limiting fibroblast proliferation and migration [113].

Tryptophan metabolites also engage the aryl hydrocarbon receptor (AhR) pathway. FICZ, an endogenous AhR ligand, increases regulatory T cells, reduces proinflammatory T cell responses, and ameliorates fibrosis in BLM-induced models [115]. Interestingly, ITE, another AhR ligand, inhibits TGF-β1–induced myofibroblast differentiation through an AhR-independent mechanism by blocking Smad2/3/4 nuclear translocation [116]. The kynurenine pathway (KP), which catabolizes over 95% of tryptophan via indoleamine 2,3-dioxygenase 1 (IDO1)/ tryptophan 2,3-dioxygenase (TDO), further modulates fibrosis. Kynurenine (Kyn), primarily produced by fibroblasts, activates AhR, induces PTEN expression, and suppresses AKT/mTOR signaling, thereby reducing fibroblast migration and activation [117]. However, TGF-β signaling can suppress AhR expression via methyl-CpG-binding domain 2 (MBD2) -mediated promoter methylation, potentially limiting the antifibrotic efficacy of Kyn [117]. Notably, Gurczynski et al. reported that in a virus-induced fibrosis model, elevated Kyn exacerbated IL-17A–dependent inflammation and collagen accumulation, suggesting that Kyn/AhR signaling may have pro-fibrotic effects under certain conditions [118].

Overall, tryptophan metabolism produces bioactive metabolites with both pro- and anti-fibrotic properties through mTOR, Smad, PI3K/AKT, and AhR-dependent pathways. These dual roles highlight the metabolic and immunologic plasticity of tryptophan in pulmonary fibrosis and suggest that selective modulation of its downstream pathways may offer promising antifibrotic strategies.

### Cysteine–GSH axis in fibrosis control

**Fig. 4:**
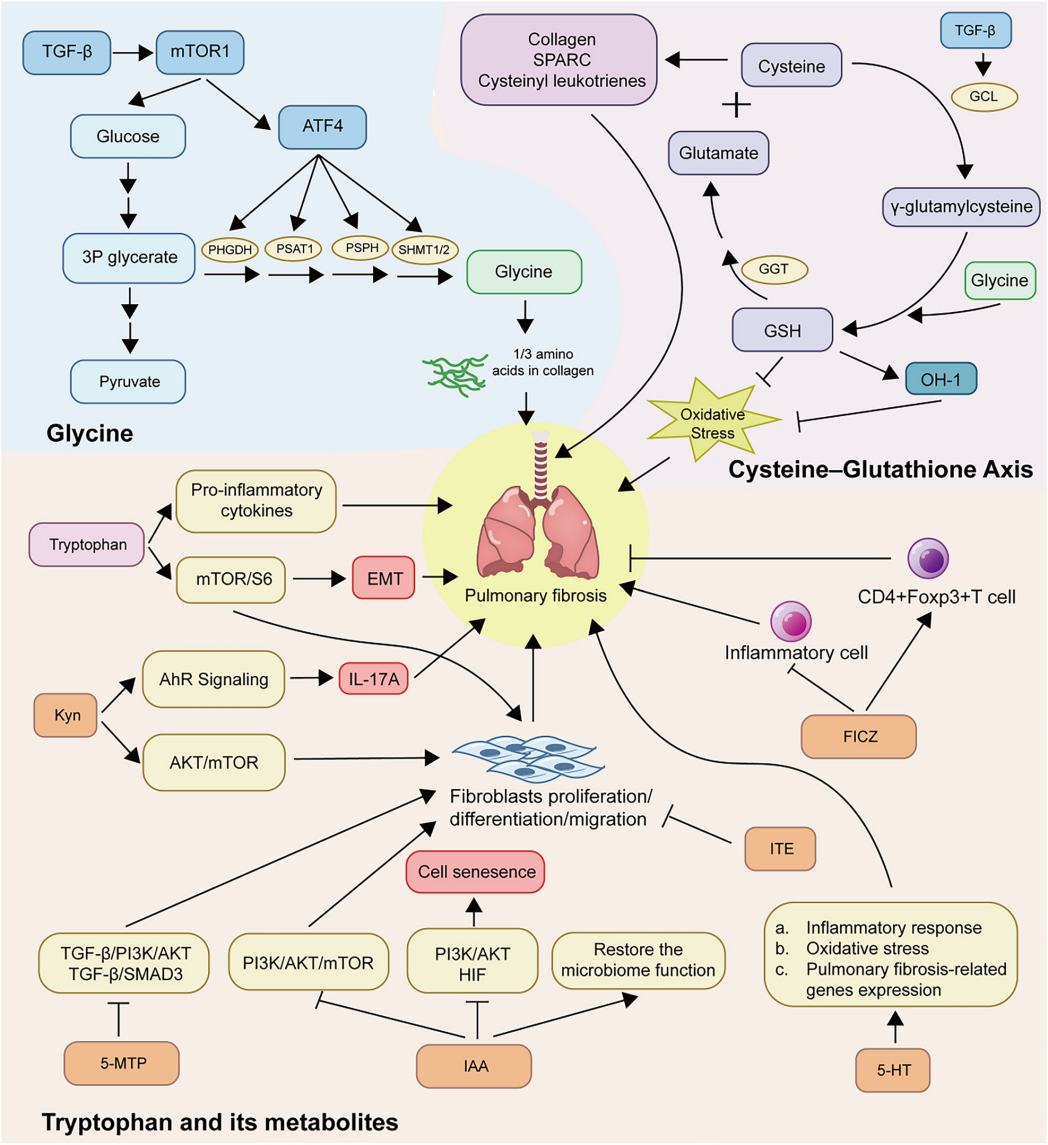
Glycine, tryptophan, and cysteine metabolism all play an important role in pulmonary fibrosis.

GSH, a key intracellular antioxidant, is synthesized from glutamate, cysteine, and glycine, with glutamate–cysteine ligase (GCL) catalyzing the rate-limiting step[119]. In IPF, GSH levels are significantly reduced, contributing to a pro-oxidant environment that promotes fibroblast proliferation and ECM accumulation [120]. Studies have shown that TGF-β1 suppresses GCL expression via the JNK–ATF3 pathway, leading to GSH depletion and enhanced oxidative stress through increased protein and lipid peroxidation [121]. Additionally, ONOO⁻-induced post-translational modifications impair the activities of both GCL and GSH synthetase, further reducing GSH synthesis [122]. Interestingly, while GSH declines in fibrosis, cysteine levels are elevated, suggesting that reduced GSH is primarily due to enzyme dysfunction rather than substrate deficiency [123]. Beyond its antioxidant role, GSH contributes to anti-fibrotic signaling. For instance, it supports the induction of heme oxygenase-1 (HO-1) in lung fibroblasts, which mitigates inflammation and fibrosis [124]. Conversely, an oxidized extracellular cysteine/cystine redox potential enhances TGF-β1 expression and stimulates fibroblast proliferation and matrix production [125]. Paradoxically, mice lacking gamma-glutamyl transpeptidase (GGT)—an enzyme essential for GSH degradation—developed milder fibrosis following BLM exposure [126]. This may be due to reduced neutrophil infiltration, matrix metalloproteinase 9 (MMP9) levels, and cysteine availability, which impaired the synthesis of collagen, secreted protein acidic and rich in cysteine (SPARC), and cysteinyl leukotrienes, all of which contribute to fibrotic remodeling [126]. These findings highlight the critical balance of GSH synthesis and cysteine redox state in regulating oxidative stress, fibroblast behavior, and fibrotic progression. Modulating the GSH-GCL axis or targeting cysteine redox signaling represents a promising therapeutic avenue for IPF (Fig. 4).

### Methionine and DAO in fibrosis regulation

Metabolomic analysis in BLM-induced pulmonary fibrosis mice revealed elevated methionine in bronchoalveolar lavage fluid (BALF) and decreased levels in serum, suggesting increased methionine and proline transport to the lungs. In IPF patient lungs, genes related to methionine transport and conversion were significantly upregulated [68]. Methionine aminopeptidase 2 (MetAP2), which removes N-terminal methionine from nascent proteins, is expressed in both healthy and fibrotic lungs. Inhibition of MetAP2 reduced fibroblast proliferation and collagen deposition in BLM-treated mice, indicating its potential as a pro-fibrotic mediator [127, 128]. In parallel, D-amino acid oxidase (DAO), an enzyme responsible for degrading D-amino acids, was found downregulated in both human IPF lungs and fibrotic mouse models. DAO deficiency (Dao−/− mice) increased susceptibility to BLM-induced fibrosis, likely due to D-amino acid accumulation disrupting metabolism, immune signaling, and epithelial homeostasis [129]. DAO also modulates type II alveolar epithelial cell senescence via the p53/p21 pathway. Reduced DAO expression enhances ATII cell proliferation, further exacerbating fibrotic remodeling [129].

## Potential applications of amino acid metabolism regulation in the diagnosis and treatment of IPF

### Amino acids as emerging biomarkers in IPF

Although traditional biomarkers such as KL-6, SP-D, MMP-7, TIMPs, and CCL18 remain commonly used in IPF diagnosis and monitoring [130], emerging evidence suggests that amino acid metabolic profiles may offer valuable diagnostic and prognostic insights. Metabolomic studies in both BLM-induced fibrosis models and IPF patients have consistently identified disturbances in amino acid pathways, particularly involving alanine, aspartate, glutamate, arginine, proline, and methionine [131]. For example, methionine levels are increased in BALF but decreased in serum, indicating potential lung-specific methionine uptake. This is supported by the upregulation of methionine metabolism-related genes in IPF lung tissues [68]. Additionally, Mendelian randomization studies have found that higher circulating glutamine is associated with reduced IPF risk, suggesting a protective role [132].

Arginine metabolism also appears relevant. Elevated fractional exhaled nitric oxide (FeNO), a byproduct of arginine conversion, has been linked to disease progression and lung function decline in IPF patients [83], indicating its potential as a non-invasive biomarker. Breathomics studies further identified increased exhaled proline, 4-hydroxyproline, and branched-chain amino acids (BCAAs: valine, leucine/isoleucine, alanine) in IPF patients [133]. These elevations are consistent with their accumulation in fibrotic lungs and their potential involvement in mTORC1-driven fibroblast activation [133, 134]. However, other studies reported that BCAA levels decline in advanced stages of IPF, which may reflect metabolic exhaustion or poor nutritional status [135]. In addition, cysteine, a precursor for GSH synthesis, was significantly elevated in fibrotic lung tissue. Advanced imaging studies using fluorescent and photoacoustic probes suggest that cysteine may serve as a promising target for non-invasive IPF diagnosis [122, 123].

Collectively, these findings highlight the potential of amino acids such as arginine, proline, glutamate, methionine, cysteine, glutamine, and BCAAs as novel biomarkers for IPF diagnosis, disease staging, and progression monitoring. Further clinical validation is needed to confirm their utility and facilitate their integration into future diagnostic protocols.

### Amino acids as therapeutic targets for IPF

#### Targeting arginine–proline metabolism in IPF

Targeting arginine and proline metabolism has emerged as a promising therapeutic strategy for IPF. Modulating key enzymes in this pathway has been shown to suppress fibrotic signaling and reduce collagen accumulation. For example, pirfenidone alleviates transplant-related pulmonary fibrosis by downregulating arginase-1 and arginase-2 expression and activity [136]. Similarly, traditional Chinese medicines, such as Shuangshen Pingfei and Saorilao-4 Decoction, improve fibrosis by correcting arginine/proline metabolic imbalances and reducing S-adenosylmethionine levels [69, 137]. Natural compounds like Trichodelphinine can inhibit arginase-1 and OAT by targeting NADPH oxidase 4 (NOX4), thereby suppressing TGF-β/Smad signaling and collagen deposition [138]. Other arginase inhibitors, including 2(S)-amino-6-boronohexanoic acid, effectively reduce hydroxyproline accumulation and reverse allergen-induced fibrosis [139]. Network pharmacology studies have identified NOS2 as a key target of Pueraria lobata, linking its antifibrotic effects to arginine–NO signaling [13]. Additionally, Tianlongkechuanling (TL) mitigates fibrosis by reducing the expression of multiple arginine cycle enzymes, including arginase-1, CPS1, OTC, ASS1, OAT, ASL, and iNOS, helping to restore the balance between arginase and NO synthase pathways [140].

Interestingly, arginine metabolism exhibits dual roles in fibrosis. While excessive arginine conversion through the arginase–ornithine–proline axis promotes collagen synthesis and fibrosis, arginine supplementation has demonstrated anti-inflammatory effects, particularly in models of LPS-induced lung injury, by reducing cytokine production and immune cell infiltration [141]. The approved antifibrotic agent nintedanib may also influence this pathway by improving the ADMA/arginine ratio, enhancing NOS activity, restoring NO levels, and reducing oxidative stress [142]. In the proline synthesis branch, prolyl-tRNA synthetase 1 (PARS1) has been identified as a novel antifibrotic target. While the PARS1 inhibitor halofuginone exhibited toxicity, its optimized derivative DWN12088 demonstrated improved safety and efficacy in preclinical models and is currently under clinical investigation for IPF [143]. (Table 1).

**Table 1:** The clinical trials for DWN12088 to treat IPF (revealed by search via the U.S. National Library of Medicine, ClinicalTrials.gov).

Drug	Target	Trial Number	Phase	Primary Outcome Measures	Disease	Status at March 2025
DWN12088	PARS 1	NCT05389215	Phase 2	Rate of decline of FVC, Incidents of treatment-emergent adverse events	IPF	Recruiting
		NCT04888715	Phase 1	Cmax/AUC of Pirfenidone/ Nintedanib and DWN12088	IPF	Completed
		NCT04888728	Phase 1	Cmax/AUCt of Nebivolol/ DWN12088	IPF	Completed
		NCT04767815	Phase 1	Cmax/AUClast of DWN12088 and metabolite, Adverse events such as subjective and objective symptoms	IPF	Completed

Overall, the arginine–proline metabolic axis plays a complex role in IPF, modulating both fibrotic and inflammatory processes. Future therapeutic strategies should carefully balance the pro-fibrotic and anti-inflammatory functions of this pathway to achieve optimal clinical benefit.

#### Targeting glutamine transport and metabolism in IPF

In pulmonary fibrosis, the neutral amino acid transporter SLC1A5 (also known as ASCT2) plays a key role in glutamine uptake, which is critical for fibrotic progression. Studies have shown that SLC1A5 expression is upregulated in IPF and that TGF-β‘s profibrotic effects depend, in part, on its activity [144, 145]. SLC1A5 promotes the expression of fibrotic targets, cell migration, and TGF-β-dependent growth. Inhibiting SLC1A5 with small molecule inhibitors like V-9302 has been shown to significantly alleviate lung injury, fibrosis, and inflammation in BLM-induced IPF mouse models [145]. While this offers a promising treatment avenue, the effects in other clinical models remain limited, and long-term treatment toxicity requires further study.

In addition to SLC1A5, glutamine metabolism itself is a target for fibrosis treatment. GLS1, a key enzyme in glutamine metabolism, has been shown to contribute to pulmonary fibrosis. GLS1 inhibition using the small molecule CB-839 has demonstrated efficacy in alleviating BLM-induced pulmonary fibrosis [98, 100]. Moreover, Tanshinone IIA (Tan IIA), a natural compound from Salvia miltiorrhiza, has been found to have anti-fibrotic effects. It activates the Nrf2 pathway, directing glutamine catabolism toward GSH production and reducing glutamate’s role in biosynthesis. Tan IIA also inhibits GLS1 expression and decreases proline synthesis, thereby reducing collagen production in myofibroblasts [146, 147]. TGF-β induces GLS1 via the PI3K/mTORC2/PDGFR signaling pathway, promoting fibrotic target expression [100]. Targeting PI3K/mTOR pathways has shown promise in preclinical trials, with omipalisib being well-tolerated in IPF subjects [148, 149]. Additionally, sirolimus treatment reduced fibrocyte concentration in IPF patients by 35%, showing potential for PI3K/mTOR inhibition in fibrosis management [148] (Table 2).

**Table 2:** The clinical trials for inhibitors of PI3K/mTOR to treat IPF (revealed by search via the U.S. National Library of Medicine, ClinicalTrials.gov).

Drug	Target	Trial number	Phase	Disease	Status at March 2025
Omipalisib (GSK2126458)	PI3K/mTOR	NCT01725139	Phase 1	IPF	Completed
HEC 68498	PI3K/mTOR	NCT03502902	Phase 1	IPF	Completed
Sirolimus	PI3K/mTOR	NCT01462006	-	IPF	Completed
Sirolimus	PI3K/mTOR	NCT04948203	Phase 2/Phase 3	Post-OVID Fibrosis	Recruiting

In summary, targeting SLC1A5, GLS1, and glutamine metabolism presents a potential therapeutic strategy for treating pulmonary fibrosis, but further research is needed to refine these approaches.

#### Modulating tryptophan pathways in IPF

Tranilast (N-[3’,4’-dimethoxy-cinnamoyl]-o-aminobenzoic acid), a structural analog of tryptophan metabolites, has demonstrated antifibrotic potential. In smoke-induced pulmonary fibrosis models, tranilast treatment significantly reduced inflammatory cytokines (IL-1β, TNF-α, TGF-β1), oxidative stress, and NF-κB expression, while also inhibiting fibroblast proliferation in lung tissue [150]. Tryptophan and its degradation product kynurenine are known markers of systemic immune activation. Elevated kynurenine/tryptophan (Kyn/Trp) ratios are often observed in inflammatory states. Notably, pirfenidone therapy has been shown to reduce the Kyn/Trp ratio, suggesting that modulation of the Kyn-Trp pathway may contribute to its antifibrotic effects [142].

#### NAC and SAC in antioxidant-based IPF therapy

N-acetyl-L-cysteine (NAC), a precursor to L-cysteine and key substrate in GSH synthesis, exhibits strong antioxidant properties. NAC has been shown to eliminate ROS and suppress TGF-β1–induced profibrotic responses in lung fibroblasts [19, 151, 152]. In vivo, it reduces hydroxyproline, TGF-β, and IL-17 levels, highlighting its anti-fibrotic and anti-inflammatory potential [153]. Nanoparticle modification, such as combining NAC with nintedanib-loaded chitosan, has enhanced its anti-fibrotic effect without harming normal cells [154]. Clinical trials on NAC in IPF have yielded mixed results: some showed slowed lung function decline [155], while a large phase 3 trial found no significant benefit in preserving forced vital capacity (FVC) in patients with mild-to-moderate IPF [156]. Furthermore, combination therapy with prednisone, azathioprine, and NAC was associated with increased mortality and hospitalization, indicating potential risks with polypharmacy [157] (Table 3).

**Table 3:** The clinical trials for NAC to treat IPF(revealed by search via the U.S. National Library of Medicine, ClinicalTrials.gov).

Drug	Trial number	Phase	Primary outcome measures	Disease	Status at March 2025
NAC	NCT03720483	Phase 1 and 2	Changes in Pulmonary function-forced vital capacity (FVC), diffusion capacity for CO (DLCO)	IPF	Withdrawn
NAC	NCT00639496	Phase 3	Vital capacity (VC) and DLCO	IPF	Completed
NAC	NCT04300920	Phase 3	Time to one of the following composite endpoint criteria: 10% relative decline in FVC, first respiratory hospitalization, lung transplant or death from any cause	IPF	Active, not recruiting
Prednisone, Azathioprine, and NAC	NCT00650091	Phase 3	Overall Change in FVC	IPF	Completed
NAC	NCT02707640	Phase 2	Percentage of participants with dose reductions, early treatment discontinuations, treatment-emergent adverse events (TEAEs), treatment-emergent serious adverse events (SAEs), treatment-emergent adverse events resulting in permanent discontinuation of study treatment, treatment-emergent deaths of all causes, treatment-emergent adverse events that led to dose reduction or temporary discontinuation of study treatment	IPF	Completed

Another cysteine-derived compound, S-allyl-L-cysteine (SAC), has demonstrated superior antifibrotic activity. SAC inhibits TGF-β1–induced myofibroblast differentiation, downregulates AKT/NF-κB signaling, and preserves thiol homeostasis. In experimental models, SAC outperformed both NAC and L-cysteine by reducing oxidative stress, leukocyte infiltration, and iNOS expression, making it a promising candidate for fibrosis prevention [158–160].

#### Modulation of amino acid metabolism by pharmacological and bioactive agents in IPF

Recent studies have highlighted that pharmacological interventions and certain bioactive compounds can reprogram amino acid metabolism, offering potential therapeutic benefits in pulmonary fibrosis. For instance, chlorquinaldol, a known antimicrobial agent, was shown to activate the methionine cycle through methionine synthase reductase (MTRR), thereby increasing methionine and S-adenosylmethionine levels and ultimately suppressing fibroblast activation [161]. The Phosphodiesterase (PDE) 4 inhibitor roflumilast, which is clinically approved for chronic obstructive pulmonary disease (COPD), has been reported to reverse BLM-induced elevations in glycine and proline and moderately restore levels of glutamate, alanine, and arginine. However, it did not significantly affect BLM-induced protein synthesis [162].

In addition to synthetic drugs, several natural bioactive compounds and dietary interventions have been found to influence amino acid metabolism during fibrotic progression. For example, sea cucumber-derived peptides significantly downregulated amino acids such as N-acetyl-L-histidine, L-threonine, L-alanine, and glycine in fibrotic models, suggesting their potential role in metabolic remodeling and fibrosis resolution [163]. Similarly, Amygdalus mongolica oil, a plant-derived extract, increased L-serine and citrulline levels, which are associated with anti-inflammatory and antioxidant functions, and upregulated genes involved in oxidative stress defense [164].

Prismatomeris connata extract was shown to attenuate BLM-induced pulmonary fibrosis, likely through suppression of the TGF-β1/Smad signaling pathway. Spatial metabolomics analysis suggested its antifibrotic effect may be partially attributed to modulation of arginine biosynthesis and the metabolism of alanine, aspartate, and glutamate [131]. Furthermore, in a paraquat-induced fibrosis model, Xuebijing injection, a traditional Chinese medicine compound, corrected elevated levels of L-valine, glycine, and L-tryptophan, indicating its antifibrotic potential may be related to rebalancing amino acid metabolic disturbances [165].

Overall, while some of these agents (such as roflumilast and chlorquinaldol) are existing drugs, many others, including plant extracts and peptides, are better described as bioactive compounds with metabolic regulatory effects rather than conventional pharmacological therapies. Their mechanisms of action suggest a promising avenue for future antifibrotic interventions, but their clinical translation will require further validation.

## Summary and perspectives

This review highlights the pivotal role of amino acid metabolism in the pathogenesis of pulmonary fibrosis, with a focus on how dysregulated metabolic pathways—particularly those involving proline, arginine, glutamine, and tryptophan—drive collagen deposition, fibroblast activation, and disease progression. Growing evidence indicates that targeting specific amino acid metabolic pathways holds promise as a novel therapeutic strategy for pulmonary fibrosis. While the precise mechanisms linking these metabolic alterations to fibrotic remodeling remain to be fully elucidated, current studies strongly support amino acid metabolism as a central regulatory axis in fibrosis initiation and progression. Advances in metabolomics and molecular biology are expected to further uncover the complex interplay between metabolism and fibrosis, offering new opportunities for biomarker discovery, early diagnosis, and the development of targeted, personalized treatments for pulmonary fibrosis.

## References

[1] Lederer DJ, Longo DL, Martinez FJ. Idiopathic pulmonary fibrosis. N Engl J Med. 2018;378:1811–23. 10.1056/NEJMra1705751. 29742380 10.1056/NEJMra1705751

[2] Nakamura Y, Suda T. Idiopathic pulmonary fibrosis: diagnosis and clinical manifestations. Clin Med Insights Circ Respir Pulm Med. 2015;9:163–71. 10.4137/CCRPM.S39897. 27625576 10.4137/CCRPM.S39897 PMC5013866

[3] Podolanczuk AJ, Thomson, CC, Remy-Jardin M, Richeldi L, Martinez FJ, Kolb M, et al. Idiopathic pulmonary fibrosis: state of the art for 2023. Eur Respir J. 2023;61. 10.1183/13993003.00957-2022. 10.1183/13993003.00957-2022 36702498

[4] Raghu G, Chen S-Y, Yeh W-S, Maroni B, Li Q, Lee Y-C, et al. Idiopathic pulmonary fibrosis in US Medicare beneficiaries aged 65 years and older: incidence, prevalence, and survival, 2001–11. Lancet Respir Med. 2014;2:566–72. 10.1016/S2213-2600(14)70101-8. 24875841 10.1016/S2213-2600(14)70101-8

[5] George PM, Patterson CM, Reed AK, Thillai M. Lung transplantation for idiopathic pulmonary fibrosis. Lancet Respir Med. 2019;7:271–82. 10.1016/s2213-2600(18)30502-2. 30738856 10.1016/S2213-2600(18)30502-2

[6] Wilson MS, Wynn TA. Pulmonary fibrosis: pathogenesis, etiology and regulation. Mucosal Immunol. 2009;2:103–21. 10.1038/mi.2008.85. 19129758 10.1038/mi.2008.85 PMC2675823

[7] Chandel NS. Amino acid metabolism. Cold Spring Harb Perspect Biol. 2021;13. 10.1101/cshperspect.a040584. 10.1101/cshperspect.a040584 PMC8015690 33795250

[8] Ling ZN, Jiang YF, Ru JN, Lu JH, Ding B, Wu J. Amino acid metabolism in health and disease. Signal Transduct Target Ther. 2023;8:345 10.1038/s41392-023-01569-3. 37699892 10.1038/s41392-023-01569-3 PMC10497558

[9] Li X, Zhang HS. Amino acid metabolism, redox balance and epigenetic regulation in cancer. Febs j. 2024;291:412–29. 10.1111/febs.16803. 37129434 10.1111/febs.16803

[10] Bröer S. Intestinal amino acid transport and metabolic health. Annu Rev Nutr. 2023;43:73–99. 10.1146/annurev-nutr-061121-094344. 37285555 10.1146/annurev-nutr-061121-094344

[11] King TE Jr., Pardo A, Selman M. Idiopathic pulmonary fibrosis. Lancet. 2011;378:1949–61. 10.1016/S0140-6736(11)60052-4. 21719092 10.1016/S0140-6736(11)60052-4

[12] Zhao J, Yu W, Zhou D, Liu Y, Wei J, Bi L, et al. Delineating, imaging, and assessing pulmonary fibrosis remodeling via collagen hybridization. ACS Nano. 2024;18:27997–8011. 10.1021/acsnano.4c06139. 39361472 10.1021/acsnano.4c06139

[13] Du H, Shao M, Xu S, Yang Q, Xu J, Ke H, et al. Integrating metabolomics and network pharmacology analysis to explore mechanism of Pueraria lobata against pulmonary fibrosis: Involvement of arginine metabolism pathway. J Ethnopharmacol. 2024;332:118346 10.1016/j.jep.2024.118346. 38782311 10.1016/j.jep.2024.118346

[14] Zhang P, Wang J, Luo W, Yuan J, Cui C, Guo L, et al. Kindlin-2 acts as a key mediator of lung fibroblast activation and pulmonary fibrosis progression. Am J Respir Cell Mol Biol. 2021;65:54–69. 10.1165/rcmb.2020-0320OC. 33761308 10.1165/rcmb.2020-0320OC

[15] Endo M, Oyadomari S, Terasaki Y, Takeya M, Suga M, Mori M, et al. Induction of arginase I and II in bleomycin-induced fibrosis of mouse lung. Am J Physiol Lung Cell Mol Physiol. 2003;285:L313–L321. 10.1152/ajplung.00434.2002. 12679322 10.1152/ajplung.00434.2002

[16] Liu JQ, Zhou HB, Bai WF, Wang J, Li Q, Fan LY, et al. Assessment of progression of pulmonary fibrosis based on metabonomics and analysis of intestinal microbiota. Artif Cells Nanomed Biotechnol. 2024;52:201–17. 10.1080/21691401.2024.2326616. 38488151 10.1080/21691401.2024.2326616

[17] Hamanaka RB, Nigdelioglu R, Meliton AY, Tian Y, Witt LJ, O’Leary E, et al. Inhibition of phosphoglycerate dehydrogenase attenuates bleomycin-induced pulmonary fibrosis. Am J Respir Cell Mol Biol. 2018;58:585–93. 10.1165/rcmb.2017-0186OC. 29019702 10.1165/rcmb.2017-0186OC PMC5946329

[18] Nigdelioglu R, Hamanaka RB, Meliton AY, O’Leary E, Witt LJ, Cho T, et al. Transforming growth factor (TGF)-beta promotes de novo serine synthesis for collagen production. J Biol Chem. 2016;291:27239–51. 10.1074/jbc.M116.756247. 27836973 10.1074/jbc.M116.756247 PMC5207151

[19] Sugiura H, Ichikawa T, Liu X, Kobayashi T, Wang XQ, Kawasaki S, et al. N-acetyl-L-cysteine inhibits TGF-beta1-induced profibrotic responses in fibroblasts. Pulm Pharm Ther. 2009;22:487–91. 10.1016/j.pupt.2009.04.002. 10.1016/j.pupt.2009.04.002 19393328

[20] Moss BJ, Ryter SW, Rosas IO. Pathogenic mechanisms underlying idiopathic pulmonary fibrosis. Annu Rev Pathol. 2022;17:515–46. 10.1146/annurev-pathol-042320-030240. 34813355 10.1146/annurev-pathol-042320-030240

[21] Moll M, Peljto AL, Kim JS, Xu H, Debban CL, Chen X, et al. A polygenic risk score for idiopathic pulmonary fibrosis and interstitial lung abnormalities. Am J Respir Crit Care Med. 2023;208:791–801. 10.1164/rccm.202212-2257OC. 37523715 10.1164/rccm.202212-2257OC PMC10563194

[22] Leavy OC, Ma SF, Molyneaux PL, Maher TM, Oldham JM, Flores C, et al. Proportion of idiopathic pulmonary fibrosis risk explained by known common genetic loci in European populations. Am J Respir Crit Care Med. 2021;203:775–8. 10.1164/rccm.202008-3211LE. 33226834 10.1164/rccm.202008-3211LE PMC7958523

[23] Allen RJ, Porte J, Braybrooke R, Flores C, Fingerlin TE, Oldham JM, et al. Genetic variants associated with susceptibility to idiopathic pulmonary fibrosis in people of European ancestry: a genome-wide association study. Lancet Respir Med. 2017;5:869–80. 10.1016/s2213-2600(17)30387-9. 29066090 10.1016/S2213-2600(17)30387-9 PMC5666208

[24] Noth I, Zhang Y, Ma SF, Flores C, Barber M, Huang Y, et al. Genetic variants associated with idiopathic pulmonary fibrosis susceptibility and mortality: a genome-wide association study. Lancet Respir Med. 2013;1:309–17. 10.1016/s2213-2600(13)70045-6. 24429156 10.1016/S2213-2600(13)70045-6 PMC3894577

[25] Fingerlin TE, Murphy E, Zhang W, Peljto AL, Brown KK, Steele MP, et al. Genome-wide association study identifies multiple susceptibility loci for pulmonary fibrosis. Nat Genet. 2013;45:613–20. 10.1038/ng.2609. 23583980 10.1038/ng.2609 PMC3677861

[26] Mostafaei S, Sayad B, Azar MEF, Doroudian M, Hadifar S, Behrouzi A, et al. The role of viral and bacterial infections in the pathogenesis of IPF: a systematic review and meta-analysis. Respir Res. 2021;22:53 10.1186/s12931-021-01650-x. 33579274 10.1186/s12931-021-01650-x PMC7880524

[27] Sheng G, Chen P, Wei Y, Yue H, Chu J, Zhao J, et al. Viral Infection Increases the Risk of Idiopathic Pulmonary Fibrosis: A Meta-Analysis. Chest. 2020;157:1175–87. 10.1016/j.chest.2019.10.032. 31730835 10.1016/j.chest.2019.10.032 PMC7214095

[28] Zhang Y, Huang W, Zheng Z, Wang W, Yuan Y, Hong Q, et al. Cigarette smoke-inactivated SIRT1 promotes autophagy-dependent senescence of alveolar epithelial type 2 cells to induce pulmonary fibrosis. Free Radic Biol Med. 2021;166:116–27. 10.1016/j.freeradbiomed.2021.02.013. 33609723 10.1016/j.freeradbiomed.2021.02.013

[29] Wijsenbeek M, Cottin V. Spectrum of fibrotic lung diseases. N Engl J Med. 2020;383:958–68. 10.1056/NEJMra2005230. 32877584 10.1056/NEJMra2005230

[30] Distler JHW, Gyorfi AH, Ramanujam M, Whitfield ML, Konigshoff M, Lafyatis R. Shared and distinct mechanisms of fibrosis. Nat Rev Rheumatol. 2019;15:705–30. 10.1038/s41584-019-0322-7. 31712723 10.1038/s41584-019-0322-7

[31] Winters NI, Burman A, Kropski JA, Blackwell TS. Epithelial injury and dysfunction in the pathogenesis of idiopathic pulmonary fibrosis. Am J Med Sci. 2019;357:374–8. 10.1016/j.amjms.2019.01.010. 31010463 10.1016/j.amjms.2019.01.010 PMC6481315

[32] Katzenstein AL. Pathogenesis of “fibrosis” in interstitial pneumonia: an electron microscopic study. Hum Pathol. 1985;16:1015–24. 10.1016/s0046-8177(85)80279-3. 4043950 10.1016/s0046-8177(85)80279-3

[33] Ma H, Liu S, Li S, Xia Y. Targeting growth factor and cytokine pathways to treat idiopathic pulmonary fibrosis. Front Pharmacol. 2022;13:918771 10.3389/fphar.2022.918771. 35721111 10.3389/fphar.2022.918771 PMC9204157

[34] Wynn TA. Integrating mechanisms of pulmonary fibrosis. J Exp Med. 2011;208:1339–50. 10.1084/jem.20110551. 21727191 10.1084/jem.20110551 PMC3136685

[35] Liu X, Dai K, Zhang X, Huang G, Lynn H, Rabata A, et al. Multiple fibroblast subtypes contribute to matrix deposition in pulmonary fibrosis. Am J Respir Cell Mol Biol. 2023;69:45–56. 10.1165/rcmb.2022-0292OC. 36927333 10.1165/rcmb.2022-0292OC PMC10324043

[36] Peng D, Fu M, Wang M, Wei Y, Wei X. Targeting TGF-β signal transduction for fibrosis and cancer therapy. Mol Cancer. 2022;21:104. 10.1186/s12943-022-01569-x. 35461253 10.1186/s12943-022-01569-x PMC9033932

[37] Nakamura Y, Shimizu Y. Cellular and molecular control of lipid metabolism in idiopathic pulmonary fibrosis: clinical application of the lysophosphatidic acid pathway. Cells. 2023;12. 10.3390/cells12040548 10.3390/cells12040548 PMC9954511 36831215

[38] Vander Ark A, Cao J, Li X. TGF-beta receptors: In and beyond TGF-beta signaling. Cell Signal. 2018;52:112–20. 10.1016/j.cellsig.2018.09.002. 30184463 10.1016/j.cellsig.2018.09.002

[39] Deng Z, Fan T, Xiao C, Tian H, Zheng Y, Li C, et al. TGF-β signaling in health, disease, and therapeutics. Signal Transduct Target Ther. 2024;9:61 10.1038/s41392-024-01764-w. 38514615 10.1038/s41392-024-01764-w PMC10958066

[40] Tzavlaki K, Moustakas A. TGF-beta signaling. Biomolecules. 2020;10. 10.3390/biom10030487. 10.3390/biom10030487 PMC7175140 32210029

[41] Fernandez IE, Eickelberg O. The impact of TGF-beta on lung fibrosis: from targeting to biomarkers. Proc Am Thorac Soc. 2012;9:111–6. 10.1513/pats.201203-023AW. 22802283 10.1513/pats.201203-023AW

[42] Wan R, Wang L, Duan Y, Zhu M, Li W, Zhao M, et al. ADRB2 inhibition combined with antioxidant treatment alleviates lung fibrosis by attenuating TGFβ/SMAD signaling in lung fibroblasts. Cell Death Discov. 2023;9:407 10.1038/s41420-023-01702-9. 37923730 10.1038/s41420-023-01702-9 PMC10624856

[43] Huang G, Yang X, Yu Q, Luo Q, Ju C, Zhang B, et al. Overexpression of STX11 alleviates pulmonary fibrosis by inhibiting fibroblast activation via the PI3K/AKT/mTOR pathway. Signal Transduct Target Ther. 2024;9:306 10.1038/s41392-024-02011-y. 39523374 10.1038/s41392-024-02011-y PMC11551190

[44] Chen H, Chen H, Liang J, Gu X, Zhou J, Xie C, et al. TGF-β1/IL-11/MEK/ERK signaling mediates senescence-associated pulmonary fibrosis in a stress-induced premature senescence model of Bmi-1 deficiency. Exp Mol Med. 2020;52:130–51. 10.1038/s12276-019-0371-7. 31959867 10.1038/s12276-019-0371-7 PMC7000795

[45] Wilkes MC, Mitchell H, Penheiter SG, Doré JJ, Suzuki K, Edens M, et al. Transforming growth factor-beta activation of phosphatidylinositol 3-kinase is independent of Smad2 and Smad3 and regulates fibroblast responses via p21-activated kinase-2. Cancer Res. 2005;65:10431–40. 10.1158/0008-5472.Can-05-1522. 16288034 10.1158/0008-5472.CAN-05-1522

[46] Kim JH, An GH, Kim JY, Rasaei R, Kim WJ, Jin X, et al. Human pluripotent stem-cell-derived alveolar organoids for modeling pulmonary fibrosis and drug testing. Cell Death Discov. 2021;7:48 10.1038/s41420-021-00439-7. 33723255 10.1038/s41420-021-00439-7 PMC7961057

[47] Elango R. Tolerable upper intake level for individual amino acids in humans: a narrative review of recent clinical studies. Adv Nutr. 2023;14:885–94. 10.1016/j.advnut.2023.04.004. 37062432 10.1016/j.advnut.2023.04.004 PMC10334138

[48] Kelly B, Pearce EL. Amino assets: how amino acids support immunity. Cell Metab. 2020;32:154–75. 10.1016/j.cmet.2020.06.010. 32649859 10.1016/j.cmet.2020.06.010

[49] Walker MC, van der Donk WA. The many roles of glutamate in metabolism. J Ind Microbiol Biotechnol. 2016;43:419–30. 10.1007/s10295-015-1665-y. 26323613 10.1007/s10295-015-1665-y PMC4753154

[50] Hamanaka RB, Shin KWD, Atalay MV, Cetin-Atalay R, Shah H, Szafran JCH, et al. Role of arginine and its metabolism in TGF-β-induced activation of lung fibroblasts. bioRxiv. 2024. 10.1101/2024.11.01.618293.

[51] Taheri F, Ochoa JB, Faghiri Z, Culotta K, Park HJ, Lan MS, et al. L-Arginine regulates the expression of the T-cell receptor zeta chain (CD3zeta) in Jurkat cells. Clin Cancer Res. 2001;7:958s–965s. 11300497

[52] Rodriguez PC, Quiceno DG, Ochoa AC. L-arginine availability regulates T-lymphocyte cell-cycle progression. Blood. 2007;109:1568–73. 10.1182/blood-2006-06-031856. 17023580 10.1182/blood-2006-06-031856 PMC1794048

[53] Warr RG, Hawgood S, Buckley DI, Crisp TM, Schilling J, Benson BJ, et al. Low molecular weight human pulmonary surfactant protein (SP5): isolation, characterization, and cDNA and amino acid sequences. Proc Natl Acad Sci USA. 1987;84:7915–9. 10.1073/pnas.84.22.7915. 3479771 10.1073/pnas.84.22.7915 PMC299446

[54] Agassandian M, Mallampalli RK. Surfactant phospholipid metabolism. Biochim Biophys Acta. 2013;1831:612–25. 10.1016/j.bbalip.2012.09.010. 23026158 10.1016/j.bbalip.2012.09.010 PMC3562414

[55] Johansson J, Curstedt T, Robertson B. The proteins of the surfactant system. Eur Respir J. 1994;7:372–91. 10.1183/09031936.94.07020372. 8162991 10.1183/09031936.94.07020372

[56] Yu SH, Possmayer F. Comparative studies on the biophysical activities of the low-molecular-weight hydrophobic proteins purified from bovine pulmonary surfactant. Biochim Biophys Acta. 1988;961:337–50. 10.1016/0005-2760(88)90081-1. 3401500 10.1016/0005-2760(88)90081-1

[57] Lian G, Gnanaprakasam JR, Wang T, Wu R, Chen X, Liu L, et al. Glutathione de novo synthesis but not recycling process coordinates with glutamine catabolism to control redox homeostasis and directs murine T cell differentiation. Elife. 2018;7. 10.7554/eLife.36158. 10.7554/eLife.36158 PMC6152796 30198844

[58] Makena P, Kikalova T, Prasad GL, Baxter SA. Oxidative stress and lung fibrosis: towards an adverse outcome pathway. Int J Mol Sci. 2023;24 (2023). 10.3390/ijms241512490 PMC10419527 37569865

[59] MacNee W. Oxidative stress and lung inflammation in airways disease. Eur J Pharmacol. 2001;429:195–207. 10.1016/s0014-2999(01)01320-6. 11698041 10.1016/s0014-2999(01)01320-6

[60] Bargagli E, Olivieri C, Bennett D, Prasse A, Muller-Quernheim J, Rottoli P. Oxidative stress in the pathogenesis of diffuse lung diseases: a review. Respir Med. 2009;103:1245–56. 10.1016/j.rmed.2009.04.014. 19464864 10.1016/j.rmed.2009.04.014

[61] Venoji R, Amirtharaj GJ, Kini A, Vanaparthi S, Venkatraman A, Ramachandran A. Enteral glutamine differentially regulates Nrf 2 along the villus-crypt axis of the intestine to enhance glutathione levels. J Gastroenterol Hepatol. 2015;30:1740–7. 10.1111/jgh.13019. 26095579 10.1111/jgh.13019

[62] Chao YK, Wu YC, Yang KJ, Chiang LL, Liu HP, Lin PJ, et al. Pulmonary perfusion with L-arginine ameliorates post-cardiopulmonary bypass lung injury in a rabbit model. J Surg Res. 2011;167:e77–83. 10.1016/j.jss.2009.10.041. 20189593 10.1016/j.jss.2009.10.041

[63] Mabalirajan U, Ahmad T, Leishangthem GD, Dinda AK, Agrawal A, Ghosh B. L-arginine reduces mitochondrial dysfunction and airway injury in murine allergic airway inflammation. Int Immunopharmacol. 2010;10:1514–9. 10.1016/j.intimp.2010.08.025. 20840838 10.1016/j.intimp.2010.08.025

[64] Zhang Y, Ma X, Jiang D, Chen J, Jia H, Wu Z, et al. Glycine attenuates lipopolysaccharide-induced acute lung injury by regulating NLRP3 inflammasome and NRF2 signaling. Nutrients. 2020;12. 10.3390/nu12030611. 10.3390/nu12030611 PMC7146254 32110933

[65] Chen J, Jin Y, Yang Y, Wu Z, Wu G. Amino acids in nutrition and health: amino acids in systems function and health. In: Guoyao W, editor. Springer International Publishing; 2020. p. 57–70.

[66] Weckerle J, Picart-Armada S, Klee S, Bretschneider T, Luippold AH, Rist W, et al. Mapping the metabolomic and lipidomic changes in the bleomycin model of pulmonary fibrosis in young and aged mice. Dis Model Mech. 2022;15. 10.1242/dmm.049105. 10.1242/dmm.049105 PMC8807555 34845494

[67] Zhao YD, Yin L, Archer S, Lu C, Zhao G, Yao Y, et al. Metabolic heterogeneity of idiopathic pulmonary fibrosis: a metabolomic study. BMJ Open Respiratory Research. 2017;4. 10.1136/bmjresp-2017-000183. 10.1136/bmjresp-2017-000183 PMC5531310 28883924

[68] Nojima Y, Takeda Y, Maeda Y, Bamba T, Fukusaki E, Itoh MN, et al. Metabolomic analysis of fibrotic mice combined with public RNA-Seq human lung data reveal potential diagnostic biomarker candidates for lung fibrosis. FEBS Open Bio. 2020;10:2427–36. 10.1002/2211-5463.12982. 32961634 10.1002/2211-5463.12982 PMC7609803

[69] Chen Y, Li X, Fan X. Integrated proteomics and metabolomics reveal variations in pulmonary fibrosis development and the potential therapeutic effect of Shuangshen Pingfei formula. J Ethnopharmacol. 2023;303:115894 10.1016/j.jep.2022.115894. 36356715 10.1016/j.jep.2022.115894

[70] Qiu M, Qin L, Dong Y, Ma J, Yang Z, Gao Z. The study of metabolism and metabolomics in a mouse model of silica pulmonary fibrosis based on UHPLC-QE-MS. Artif Cells Nanomed Biotechnol. 2022;50:322–30. 10.1080/21691401.2022.2124517. 36433777 10.1080/21691401.2022.2124517

[71] Lee JU, Song KS, Hong J, Shin H, Park E, Baek J, et al. Role of lung ornithine aminotransferase in idiopathic pulmonary fibrosis: regulation of mitochondrial ROS generation and TGF-beta1 activity. Exp Mol Med. 2024;56:478–90. 10.1038/s12276-024-01170-w. 38413821 10.1038/s12276-024-01170-w PMC10907606

[72] Giri SN, Misra HP, Chandler DB, Chen ZL. Increases in lung prolyl hydroxylase and superoxide dismutase activities during bleomycin-induced lung fibrosis in hamsters. Exp Mol Pathol. 1983;39:317–26. 10.1016/0014-4800(83)90060-6. 6196228 10.1016/0014-4800(83)90060-6

[73] Guzy R, Redente EF. Kindlin for the fire: targeting proline synthesis to extinguish matrix production in pulmonary fibrosis. Am J Respir Cell Mol Biol. 2021;65:4–5. 10.1165/rcmb.2021-0137ED. 33844940 10.1165/rcmb.2021-0137ED PMC8320124

[74] Niese KA, Chiaramonte MG, Ellies LG, Rothenberg ME, Zimmermann N. The cationic amino acid transporter 2 is induced in inflammatory lung models and regulates lung fibrosis. Respir Res. 2010;11:87 10.1186/1465-9921-11-87. 20576117 10.1186/1465-9921-11-87 PMC2906447

[75] Kitowska K, Zakrzewicz D, Königshoff M, Chrobak I, Grimminger F, Seeger W, et al. Functional role and species-specific contribution of arginases in pulmonary fibrosis. Am J Physiol Lung Cell Mol Physiol. 2008;294:L34–L45. 10.1152/ajplung.00007.2007. 17934065 10.1152/ajplung.00007.2007

[76] Yadav P, Ortega JG, Tamaki W, Chien C, Chang KC, Biswas N, et al. Macrophage-fibroblast crosstalk drives Arg1-dependent lung fibrosis via ornithine loading. bioRxiv. 2024. 10.1101/2023.09.06.556606.

[77] Baek AR, Hong J, Song KS, Jang AS, Kim DJ, Chin SS, et al. Spermidine attenuates bleomycin-induced lung fibrosis by inducing autophagy and inhibiting endoplasmic reticulum stress (ERS)-induced cell death in mice. Exp Mol Med. 2020;52:2034–45. 10.1038/s12276-020-00545-z. 33318630 10.1038/s12276-020-00545-z PMC8080799

[78] Bassit RA, Curi R, Costa Rosa LF. Creatine supplementation reduces plasma levels of pro-inflammatory cytokines and PGE2 after a half-ironman competition. Amino Acids. 2008;35:425–31. 10.1007/s00726-007-0582-4. 17917696 10.1007/s00726-007-0582-4

[79] Fang YZ, Yang S, Wu G. Free radicals, antioxidants, and nutrition. Nutrition. 2002;18:872–9. 10.1016/s0899-9007(02)00916-4. 12361782 10.1016/s0899-9007(02)00916-4

[80] Xie D, Wang P, Chen W, Lin J, Wu M, Wang Y, et al. Urea cycle promotion via ammonia-upregulated CPS1 is involved in arsenite-induced pulmonary fibrosis through enhancing collagen synthesis. Chem Biol Interact. 2024;396:111029 10.1016/j.cbi.2024.111029. 38703806 10.1016/j.cbi.2024.111029

[81] Janssen W, Pullamsetti SS, Cooke J, Weissmann N, Guenther A, Schermuly RT. The role of dimethylarginine dimethylaminohydrolase (DDAH) in pulmonary fibrosis. J Pathol. 2012;229:242–9. 10.1002/path.4127. 10.1002/path.4127 23097221

[82] Noguchi S, Yatera K, Wang KY, Oda K, Akata K, Yamasaki K, et al. Nitric oxide exerts protective effects against bleomycin-induced pulmonary fibrosis in mice. Respir Res. 2014;15:92 10.1186/s12931-014-0092-3. 25092105 10.1186/s12931-014-0092-3 PMC4237963

[83] Cameli P, Bargagli E, Bergantini L, d’Alessandro M, Pieroni M, Fontana GA, et al. Extended exhaled nitric oxide analysis in interstitial lung diseases: a systematic review. Int J Mol Sci. 2020;21. 10.3390/ijms21176187. 10.3390/ijms21176187 PMC7503828 32867116

[84] Pullamsetti SS, Savai R, Dumitrascu R, Dahal BK, Wilhelm J, Konigshoff M, et al. The role of dimethylarginine dimethylaminohydrolase in idiopathic pulmonary fibrosis. Sci Transl Med. 2011;3:87ra53 10.1126/scitranslmed.3001725. 21677199 10.1126/scitranslmed.3001725

[85] Alhakamy NA, Alamoudi AJ, Asfour HZ, Ahmed OAA, Abdel-Naim AB, Aboubakr EM. L-arginine mitigates bleomycin-induced pulmonary fibrosis in rats through regulation of HO-1/PPAR-γ/β-catenin axis. Int Immunopharmacol. 2024;131:111834 10.1016/j.intimp.2024.111834. 38493696 10.1016/j.intimp.2024.111834

[86] Gao L, Zhang JH, Chen XX, Ren HL, Feng XL, Wang JL, et al. Combination of L-Arginine and L-Norvaline protects against pulmonary fibrosis progression induced by bleomycin in mice. Biomed Pharmacother. 2019;113:108768 10.1016/j.biopha.2019.108768. 30889486 10.1016/j.biopha.2019.108768

[87] Li JM, Chang WH, Li L, Yang DC, Hsu SW, Kenyon NJ, et al. Inositol possesses antifibrotic activity and mitigates pulmonary fibrosis. Respir Res. 2023;24:132 10.1186/s12931-023-02421-6. 37194070 10.1186/s12931-023-02421-6 PMC10189934

[88] Li JM, Yang DC, Oldham J, Linderholm A, Zhang J, Liu J, et al. Therapeutic targeting of argininosuccinate synthase 1 (ASS1)-deficient pulmonary fibrosis. Mol Ther. 2021;29:1487–1500. 10.1016/j.ymthe.2021.01.028. 33508432 10.1016/j.ymthe.2021.01.028 PMC8058484

[89] Sala L, Franco-Valls H, Stanisavljevic J, Curto J, Verges J, Pena R, et al. Abrogation of myofibroblast activities in metastasis and fibrosis by methyltransferase inhibition. Int J Cancer. 2019;145:3064–77. 10.1002/ijc.32376. 31032902 10.1002/ijc.32376

[90] Zakrzewicz D, Zakrzewicz A, Didiasova M, Korencak M, Kosanovic D, Schermuly RT, et al. Elevated protein arginine methyltransferase 1 expression regulates fibroblast motility in pulmonary fibrosis. Biochim Biophys Acta. 2015;1852:2678–88. 10.1016/j.bbadis.2015.09.008. 26391253 10.1016/j.bbadis.2015.09.008

[91] Liang J, Ran Y, Hu C, Zhou J, Ye L, Su W, et al. Inhibition of HIF-1α ameliorates pulmonary fibrosis by suppressing M2 macrophage polarization through PRMT1/STAT6 signals. International Immunopharmacology. 2025;146. 10.1016/j.intimp.2024.113931. 10.1016/j.intimp.2024.113931 39733638

[92] He H, Chen J, Zhao J, Zhang P, Qiao Y, Wan H, et al. PRMT7 targets of Foxm1 controls alveolar myofibroblast proliferation and differentiation during alveologenesis. Cell Death Dis. 2021;12:841. 10.1038/s41419-021-04129-1. 34497269 10.1038/s41419-021-04129-1 PMC8426482

[93] Yang L, Xia H, Smith K, Gilbertsen AJ, Jbeli AH, Abrahante JE, et al. Tumor suppressors RBL1 and PTEN are epigenetically silenced in IPF mesenchymal progenitor cells by a CD44/Brg1/PRMT5 regulatory complex. Am J Physiol Lung Cell Mol Physiol. 2024;327:L949–l963. 10.1152/ajplung.00182.2024. 39406384 10.1152/ajplung.00182.2024 PMC11684952

[94] Tsoyi K, Esposito AJ, Sun B, Bowen RG, Xiong K, Poli F, et al. Syndecan-2 regulates PAD2 to exert antifibrotic effects on RA-ILD fibroblasts. Sci Rep. 2022;12:2847. 10.1038/s41598-022-06678-7. 35181688 10.1038/s41598-022-06678-7 PMC8857282

[95] Martinod K, Witsch T, Erpenbeck L, Savchenko A, Hayashi H, Cherpokova D, et al. Peptidylarginine deiminase 4 promotes age-related organ fibrosis. J Exp Med. 2017;214:439–58. 10.1084/jem.20160530. 28031479 10.1084/jem.20160530 PMC5294849

[96] Panda B, Chilvery S, Devi P, Kalmegh R, Godugu C. Inhibition of peptidyl arginine deiminase-4 ameliorated pulmonary fibrosis via modulating M1/M2 polarisation of macrophages. Life Sci. 2025;362:123354 10.1016/j.lfs.2024.123354. 39755270 10.1016/j.lfs.2024.123354

[97] Ge J, Cui H, Xie N, Banerjee S, Guo S, Dubey S, et al. Glutaminolysis promotes collagen translation and stability via α-ketoglutarate–mediated mTOR activation and proline hydroxylation. Am J Respir Cell Mol Biol. 2018;58:378–90. 10.1165/rcmb.2017-0238OC. 29019707 10.1165/rcmb.2017-0238OC PMC5854958

[98] Cui H, Xie N, Jiang D, Banerjee S, Ge J, Sanders YY, et al. Inhibition of glutaminase 1 attenuates experimental pulmonary fibrosis. Am J Respir Cell Mol Biol. 2019;61:492–500. 10.1165/rcmb.2019-0051OC. 30943369 10.1165/rcmb.2019-0051OC PMC6775943

[99] Contento G, Wilson JA, Selvarajah B, Plate M, Guillotin D, Morales V, et al. Pyruvate metabolism dictates fibroblast sensitivity to GLS1 inhibition during fibrogenesis. JCI Insight. 2024;9. 10.1172/jci.insight.178453. 10.1172/jci.insight.178453 PMC11457851 39315548

[100] Choudhury M, Yin X, Schaefbauer KJ, Kang JH, Roy B, Kottom TJ, et al. SIRT7-mediated modulation of glutaminase 1 regulates TGF-β-induced pulmonary fibrosis. FASEB J. 2020;34:8920–40. 10.1096/fj.202000564R. 32519817 10.1096/fj.202000564R

[101] Wang S, Li X, Ma Q, Wang Q, Wu J, Yu H, et al. Glutamine metabolism is required for alveolar regeneration during lung injury. Biomolecules. 2022;12. 10.3390/biom12050728. 10.3390/biom12050728 PMC9138637 35625656

[102] Li G, Xu Q, Cheng D, Sun W, Liu Y, Ma D, et al. Caveolin-1 and Its Functional Peptide CSP7 Affect Silica-Induced Pulmonary Fibrosis by Regulating Fibroblast Glutaminolysis. Toxicol Sci. 2022;190:41–53. 10.1093/toxsci/kfac089. 36053221 10.1093/toxsci/kfac089

[103] Xiang Z, Bai L, Zhou JQ, Cevallos RR, Sanders JR, Liu G, et al. Epigenetic regulation of IPF fibroblast phenotype by glutaminolysis. Mol Metab. 2023;67:101655 10.1016/j.molmet.2022.101655. 36526153 10.1016/j.molmet.2022.101655 PMC9827063

[104] Bai L, Bernard K, Tang X, Hu M, Horowitz JC, Thannickal VJ, et al. Glutaminolysis epigenetically regulates antiapoptotic gene expression in idiopathic pulmonary fibrosis fibroblasts. Am J Respir Cell Mol Biol. 2019;60:49–57. 10.1165/rcmb.2018-0180OC. 30130138 10.1165/rcmb.2018-0180OC PMC6348715

[105] Li W, Qiu X, Wang J, Li H, Sun Y, Zhang F, et al. The therapeutic efficacy of glutamine for rats with smoking inhalation injury. Int Immunopharmacol. 2013;16:248–53. 10.1016/j.intimp.2013.02.022. 23499678 10.1016/j.intimp.2013.02.022

[106] Shaghaghi H, Para R, Tran C, Roman J, Ojeda-Lassalle Y, Sun J, et al. Glutamine restores mitochondrial respiration in bleomycin-injured epithelial cells. Free Radic Biol Med. 2021;176:335–44. 10.1016/j.freeradbiomed.2021.10.006. 34634441 10.1016/j.freeradbiomed.2021.10.006 PMC9121335

[107] Wang L, Huang W, Zhang L, Chen Q, Zhao H. Molecular pathogenesis involved in human idiopathic pulmonary fibrosis based on an integrated microRNA‑mRNA interaction network. Mol Med Rep. 2018;18:4365–73. 10.3892/mmr.2018.9456. 30221703 10.3892/mmr.2018.9456 PMC6172385

[108] Selvarajah B, Azuelos I, Platé M, Guillotin D, Forty EJ, Contento G, et al. mTORC1 amplifies the ATF4-dependent de novo serine-glycine pathway to supply glycine during TGF-β1–induced collagen biosynthesis. Sci Signal. 2019;12:eaav3048 10.1126/scisignal.aav3048. 31113850 10.1126/scisignal.aav3048 PMC6584619

[109] O’Leary EM, Tian Y, Nigdelioglu R, Witt LJ, Cetin-Atalay R, Meliton AY, et al. TGF-beta promotes metabolic reprogramming in lung fibroblasts via mTORC1-dependent ATF4 activation. Am J Respir Cell Mol Biol. 2020;63:601–12. 10.1165/rcmb.2020-0143OC. 32668192 10.1165/rcmb.2020-0143OC PMC7605163

[110] Sun W, Zhou S, Peng L, Wang W, Liu Y, Wang T, et al. Fatty acid oxidation-glycolysis metabolic transition affects ECM homeostasis in silica-induced pulmonary fibrosis. Adv Sci. 2024:e2407134. 10.1002/advs.202407134. 10.1002/advs.202407134 PMC11831484 39721015

[111] Shin KWD, Atalay MV, Cetin-Atalay R, O’Leary EM, Glass ME, Szafran JCH, et al. ATF4 and mTOR regulate metabolic reprogramming in TGF-beta-treated lung fibroblasts. bioRxiv. 2024. 10.1101/2024.06.12.598694.

[112] Li J, Wu W, Kong X, Yang X, Li K, Jiang Z, et al. Roles of gut microbiome-associated metabolites in pulmonary fibrosis by integrated analysis. NPJ Biofilms Microbiomes. 2024;10:154. 10.1038/s41522-024-00631-4. 39702426 10.1038/s41522-024-00631-4 PMC11659409

[113] Fang L, Chen H, Kong R, Que J. Endogenous tryptophan metabolite 5-Methoxytryptophan inhibits pulmonary fibrosis by downregulating the TGF-beta/SMAD3 and PI3K/AKT signaling pathway. Life Sci. 2020;260:118399 10.1016/j.lfs.2020.118399. 32918977 10.1016/j.lfs.2020.118399

[114] Zhuo J, Liu D, Yu Q, Hu M, Huang H, Chen Y, et al. Indole-3-acetic acid attenuates pulmonary fibrosis by modulating lung microbiota, inhibiting fibroblast activation, and alleviating alveolar epithelial cell senescence. Life Sci. 2024;359:123191 10.1016/j.lfs.2024.123191. 39481838 10.1016/j.lfs.2024.123191

[115] Takei H, Yasuoka H, Yoshimoto K, Takeuchi T. Aryl hydrocarbon receptor signals attenuate lung fibrosis in the bleomycin-induced mouse model for pulmonary fibrosis through increase of regulatory T cells. Arthritis Res Ther. 2020;22:20 10.1186/s13075-020-2112-7. 32033616 10.1186/s13075-020-2112-7 PMC7006193

[116] Lehmann GM, Xi X, Kulkarni AA, Olsen KC, Pollock SJ, Baglole CJ, et al. The aryl hydrocarbon receptor ligand ITE inhibits TGFbeta1-induced human myofibroblast differentiation. Am J Pathol. 2011;178:1556–67. 10.1016/j.ajpath.2010.12.025. 21406171 10.1016/j.ajpath.2010.12.025 PMC3078465

[117] Wang Y, Wu GR, Yue H, Zhou Q, Zhang L, He L, et al. Kynurenine acts as a signaling molecule to attenuate pulmonary fibrosis by enhancing the AHR-PTEN axis. J Adv Res. 2024. 10.1016/j.jare.2024.06.017. 10.1016/j.jare.2024.06.017 PMC12126728 38906325

[118] Gurczynski SJ, Pereira NL, Hrycaj SM, Wilke C, Zemans RL, Moore BB. Stem cell transplantation uncovers TDO-AHR regulation of lung dendritic cells in herpesvirus-induced pathology. JCI Insight. 2021;6. 10.1172/jci.insight.139965. 10.1172/jci.insight.139965 PMC7934859 33491663

[119] Wang L, Ahn YJ, Asmis R. Sexual dimorphism in glutathione metabolism and glutathione-dependent responses. Redox Biol. 2020;31:101410 10.1016/j.redox.2019.101410. 31883838 10.1016/j.redox.2019.101410 PMC7212491

[120] Cantin AM, Larivée P, Bégin RO. Extracellular glutathione suppresses human lung fibroblast proliferation. Am J Respir Cell Mol Biol. 1990;3:79–85. 10.1165/ajrcmb/3.1.79. 2363938 10.1165/ajrcmb/3.1.79

[121] Liu RM, Vayalil PK, Ballinger C, Dickinson DA, Huang WT, Wang S, et al. Transforming growth factor β suppresses glutamate-cysteine ligase gene expression and induces oxidative stress in a lung fibrosis model. Free Radic Biol Med. 2012;53:554–63. 10.1016/j.freeradbiomed.2012.05.016. 22634145 10.1016/j.freeradbiomed.2012.05.016 PMC3432394

[122] Wang H, Nie J, Li P, Zhang X, Wang Y, Zhang W, et al. Exploring idiopathic pulmonary fibrosis biomarker by simultaneous two-photon fluorescence imaging of cysteine and peroxynitrite. Anal Chem. 2022;94:11272–81. 10.1021/acs.analchem.2c01866. 35924865 10.1021/acs.analchem.2c01866

[123] Wang H, Zhang Y, Yang Y, He Z, Wu C, Zhang W, et al. In situ photoacoustic imaging of cysteine to reveal the mechanism of limited GSH synthesis in pulmonary fibrosis. Chem Commun. 2019;55:9685–8. 10.1039/c9cc03814k. 10.1039/c9cc03814k 31347620

[124] Ferguson HE, Thatcher TH, Olsen KC, Garcia-Bates TM, Baglole CJ, Kottmann RM, et al. Peroxisome proliferator-activated receptor-gamma ligands induce heme oxygenase-1 in lung fibroblasts by a PPARgamma-independent, glutathione-dependent mechanism. Am J Physiol Lung Cell Mol Physiol. 2009;297:L912–919. 10.1152/ajplung.00148.2009. 19734319 10.1152/ajplung.00148.2009 PMC2777492

[125] Ramirez A, Ramadan B, Ritzenthaler JD, Rivera HN, Jones DP, Roman J. Extracellular cysteine/cystine redox potential controls lung fibroblast proliferation and matrix expression through upregulation of transforming growth factor-beta. Am J Physiol Lung Cell Mol Physiol. 2007;293:L972–981. 10.1152/ajplung.00010.2007. 17644756 10.1152/ajplung.00010.2007

[126] Pardo A, Ruiz V, Arreola JL, Ramírez R, Cisneros-Lira J, Gaxiola M, et al. Bleomycin-induced pulmonary fibrosis is attenuated in gamma-glutamyl transpeptidase-deficient mice. Am J Respir Crit Care Med. 2003;167:925–32. 10.1164/rccm.200209-1007OC. 12468440 10.1164/rccm.200209-1007OC

[127] Datta B. MAPs and POEP of the roads from prokaryotic to eukaryotic kingdoms. Biochimie. 2000;82:95–107. 10.1016/s0300-9084(00)00383-7. 10727764 10.1016/s0300-9084(00)00383-7

[128] Kass D, Bridges RS, Borczuk A, Greenberg S. Methionine Aminopeptidase-2 as a Selective Target of Myofibroblasts in Pulmonary Fibrosis. Am J Respir Cell Mol Biol. 2007;37:193–201. 10.1165/rcmb.2006-0352OC. 17446530 10.1165/rcmb.2006-0352OC

[129] Guo X, Xu K, Wang L, Ding L, Li W, Zhang X, et al. Triiodothyronine acts on DAO to regulate pulmonary fibrosis progression by facilitating cell senescence through the p53/p21 signaling pathway. Front Pharm. 2024;15:1433186 10.3389/fphar.2024.1433186. 10.3389/fphar.2024.1433186 PMC11422212 39323641

[130] Zhu W, Liu C, Tan C, Zhang J. Predictive biomarkers of disease progression in idiopathic pulmonary fibrosis. Heliyon. 2024;10:e23543 10.1016/j.heliyon.2023.e23543. 38173501 10.1016/j.heliyon.2023.e23543 PMC10761784

[131] Jiang H, Zheng B, Hu G, Kuang L, Zhou T, Li S, et al. Spatially resolved metabolomics visualizes heterogeneous distribution of metabolites in lung tissue and the anti-pulmonary fibrosis effect of Prismatomeris connate extract. J Pharm Anal. 2024;14. 10.1016/j.jpha.2024.100971. 10.1016/j.jpha.2024.100971 PMC11459407 39381647

[132] Xu T, Liu C, Ning X, Gao Z, Li A, Wang S, et al. Causal relationship between circulating glutamine levels and idiopathic pulmonary fibrosis: a two-sample mendelian randomization study. BMC Pulmonary Med. 2024;24. 10.1186/s12890-024-03275-4. 10.1186/s12890-024-03275-4 PMC11401390 39272013

[133] Gaugg MT, Engler A, Bregy L, Nussbaumer-Ochsner Y, Eiffert L, Bruderer T, et al. Molecular breath analysis supports altered amino acid metabolism in idiopathic pulmonary fibrosis. Respirology. 2019;24:437–44. 10.1111/resp.13465. 30681243 10.1111/resp.13465

[134] Saxton RA, Sabatini DM. mTOR Signaling in Growth, Metabolism, and Disease. Cell. 2017;168:960–76. 10.1016/j.cell.2017.02.004. 28283069 10.1016/j.cell.2017.02.004 PMC5394987

[135] Summer R, Todd JL, Neely ML, Lobo LJ, Namen A, Newby LK, et al. Circulating metabolic profile in idiopathic pulmonary fibrosis: data from the IPF-PRO Registry. Respir Res. 2024;25:58 10.1186/s12931-023-02644-7. 38273290 10.1186/s12931-023-02644-7 PMC10809477

[136] Liu H, Drew P, Gaugler AC, Cheng Y, Visner GA. Pirfenidone inhibits lung allograft fibrosis through L-arginine-arginase pathway. Am J Transpl. 2005;5:1256–63. 10.1111/j.1600-6143.2005.00876.x. 10.1111/j.1600-6143.2005.00876.x 15888029

[137] Liu J, Song X, Fu X, Niu S, Chang H, Shi S, et al. Exploring the mechanism of action and potential targets of saorilao-4 decoction in the treatment of pulmonary fibrosis in rats by metabolomics. Food Sci Nutr. 2025;13:e4633 10.1002/fsn3.4633. 39898125 10.1002/fsn3.4633 PMC11783149

[138] Liu F, Yao Y, Guo C, Dai P, Huang J, Peng P, et al. Trichodelphinine A alleviates pulmonary fibrosis by inhibiting collagen synthesis via NOX4-ARG1/TGF-beta signaling pathway. Phytomedicine. 2024;132:155755 10.1016/j.phymed.2024.155755. 38870750 10.1016/j.phymed.2024.155755

[139] Maarsingh H, Dekkers BG, Zuidhof AB, Bos IS, Menzen MH, Klein T, et al. Increased arginase activity contributes to airway remodelling in chronic allergic asthma. Eur Respir J. 2011;38:318–28. 10.1183/09031936.00057710. 21310883 10.1183/09031936.00057710

[140] Zhang L, Qu S, Wang L, WangC, Yu Q, Zhang Z, et al. Tianlongkechuanling inhibits pulmonary fibrosis through down-regulation of arginase-ornithine pathway. Front Pharmacol. 2021;12. 10.3389/fphar.2021.661129. 10.3389/fphar.2021.661129 PMC8114272 33995084

[141] Ma X, Zhang Y, Jiang D, Yang Y, Wu G, Wu Z. Protective effects of functional amino acids on apoptosis, inflammatory response, and pulmonary fibrosis in lipopolysaccharide-challenged mice. J Agric Food Chem. 2019;67:4915–22. 10.1021/acs.jafc.9b00942. 31001980 10.1021/acs.jafc.9b00942

[142] Fois AG, Sotgiu E, Scano V, Negri S, Mellino S, Zinellu E, et al. Effects of pirfenidone and nintedanib on markers of systemic oxidative stress and inflammation in patients with idiopathic pulmonary fibrosis: a preliminary report. Antioxidants. 2020;9. 10.3390/antiox9111064. 10.3390/antiox9111064 PMC7692317 33143144

[143] Yoon I, Kim S, Cho M, You KA, Son J, Lee C, et al. Control of fibrosis with enhanced safety via asymmetric inhibition of prolyl-tRNA synthetase 1. EMBO Mol Med. 2023;15:e16940 10.15252/emmm.202216940. 37212275 10.15252/emmm.202216940 PMC10331583

[144] Lian N, Jin H, Zhu W, Zhang C, Qi Y, Jiang M, et al. Inhibition of glutamine transporter ASCT2 mitigates bleomycin-induced pulmonary fibrosis in mice. Acta Histochem. 2022;124:151961 10.1016/j.acthis.2022.151961. 36265204 10.1016/j.acthis.2022.151961

[145] Choudhury M, Schaefbauer KJ, Kottom TJ, Yi ES, Tschumperlin DJ, Limper AH. Targeting pulmonary fibrosis by SLC1A5-dependent glutamine transport blockade. Am J Respir Cell Mol Biol. 2023;69:441–55. 10.1165/rcmb.2022-0339OC. 37459644 10.1165/rcmb.2022-0339OC PMC10557918

[146] An L, Peng LY, Sun NY, Yang YL, Zhang XW, Li B, et al. Tanshinone IIA activates nuclear factor-erythroid 2-related factor 2 to restrain pulmonary fibrosis via regulation of redox homeostasis and glutaminolysis. Antioxid Redox Signal. 2019;30:1831–48. 10.1089/ars.2018.7569. 30105924 10.1089/ars.2018.7569

[147] Shan B, Guo C, Zhou H, Chen J. Tanshinone IIA alleviates pulmonary fibrosis by modulating glutamine metabolic reprogramming based on [U-(13)C(5)]-glutamine metabolic flux analysis. J Adv Res. 2024. 10.1016/j.jare.2024.04.029. 10.1016/j.jare.2024.04.029 PMC11976427 38697470

[148] Gomez-Manjarres, DC, Axell-House DB, Patel DC, Odackal J, Yu V, Burdick MD, et al. Sirolimus suppresses circulating fibrocytes in idiopathic pulmonary fibrosis in a randomized controlled crossover trial. JCI Insight. 2023;8. 10.1172/jci.insight.166901. 10.1172/jci.insight.166901 PMC10243828 36853800

[149] Mercer PF, Woodcock HV, Eley JD, Plate M, Sulikowski MG, Durrenberger PF, et al. Exploration of a potent PI3 kinase/mTOR inhibitor as a novel anti-fibrotic agent in IPF. Thorax. 2016;71:701–11. 10.1136/thoraxjnl-2015-207429. 27103349 10.1136/thoraxjnl-2015-207429 PMC4975851

[150] Cui P, Tang Z, Zhan Q, Deng C, Lai Y, Zhu F, et al. In vitro and vivo study of tranilast protects from acute respiratory distress syndrome and early pulmonary fibrosis induced by smoke inhalation. Burns. 2022;48:880–95. 10.1016/j.burns.2022.03.010. 35410697 10.1016/j.burns.2022.03.010

[151] Rushworth GF, Megson IL. Existing and potential therapeutic uses for N-acetylcysteine: the need for conversion to intracellular glutathione for antioxidant benefits. Pharm Ther. 2014;141:150–9. 10.1016/j.pharmthera.2013.09.006. 10.1016/j.pharmthera.2013.09.006 24080471

[152] Zheng F, Wu X, Zhang J, Fu Z, Zhang Y. Sevoflurane reduces lipopolysaccharide-induced apoptosis and pulmonary fibrosis in the RAW264.7 cells and mice models to ameliorate acute lung injury by eliminating oxidative damages. Redox Rep. 2022;27:139–49. 10.1080/13510002.2022.2096339. 35801580 10.1080/13510002.2022.2096339 PMC9272930

[153] Maghsadi Z, Azadmehr A, Moghadamnia AA, Feizi F, Hamidi N. N-Acetylcysteine attenuated pulmonary fibrosis induced by bleomycin via immunomodulation responses. Res Pharm Sci. 2023;18:177–84. 10.4103/1735-5362.367796. 36873280 10.4103/1735-5362.367796 PMC9976053

[154] Singh S, Wairkar S. Long-circulating thiolated chitosan nanoparticles of nintedanib with N-acetyl cysteine for treating idiopathic pulmonary fibrosis: In vitro assessment of cytotoxicity, antioxidant, and antifibrotic potential. Int J Pharm. 2023;644:123322 10.1016/j.ijpharm.2023.123322. 37591474 10.1016/j.ijpharm.2023.123322

[155] Behr J, Demedts M, Buhl R, Costabel U, Dekhuijzen RP, Jansen HM, et al. Lung function in idiopathic pulmonary fibrosis-extended analyses of the IFIGENIA trial. Respir Res. 2009;10:101 10.1186/1465-9921-10-101. 19860915 10.1186/1465-9921-10-101 PMC2774307

[156] Martinez FJ, de Andrade JA, Anstrom KJ, King TE Jr., Raghu G. Randomized trial of acetylcysteine in idiopathic pulmonary fibrosis. N Engl J Med. 2014;370:2093–101. 10.1056/NEJMoa1401739. 24836309 10.1056/NEJMoa1401739 PMC4116664

[157] Raghu G, Anstrom KJ, King TE Jr, Lasky JA, Martinez FJ. Prednisone, azathioprine, and N-acetylcysteine for pulmonary fibrosis. N Engl J Med. 2012;366:1968–77. 10.1056/NEJMoa1113354. 22607134 10.1056/NEJMoa1113354 PMC3422642

[158] Tsukioka T, Takemura S, Minamiyama Y, Mizuguchi S, Toda M, Okada S. Attenuation of bleomycin-induced pulmonary fibrosis in rats with S-allyl cysteine. Molecules. 2017;22. 10.3390/molecules22040543. 10.3390/molecules22040543 PMC6154609 28353632

[159] Nie Y, Yu K, Li B, Hu Y, Zhang H, Xin R, et al. S-allyl-l-cysteine attenuates bleomycin-induced pulmonary fibrosis and inflammation via AKT/NF-κB signaling pathway in mice. J Pharmacol Sci. 2019;139:377–84. 10.1016/j.jphs.2019.03.002. 30928090 10.1016/j.jphs.2019.03.002

[160] Mizuguchi S, Takemura S, Minamiyama Y, Kodai S, Tsukioka T, Inoue K, et al. S-allyl cysteine attenuated CCl4-induced oxidative stress and pulmonary fibrosis in rats. Biofactors. 2006;26:81–92. 10.1002/biof.5520260108. 16614485 10.1002/biof.5520260108

[161] Yang X, Lin G, Chen Y, Lei X, Ou Y, Yan Y, et al. Chlorquinaldol alleviates lung fibrosis in mice by inhibiting fibroblast activation through targeting methionine synthase reductase. ACS Cent Sci. 2024;10:1789–802. 10.1021/acscentsci.4c00798. 39345816 10.1021/acscentsci.4c00798 PMC11428390

[162] Milara J, Morcillo E, Monleon D, Tenor H, Cortijo J. Roflumilast prevents the metabolic effects of bleomycin-induced fibrosis in a murine model. PLoS ONE. 2015;10:e0133453 10.1371/journal.pone.0133453. 26192616 10.1371/journal.pone.0133453 PMC4507994

[163] Wu J, Chen Y, Zhang J, Cheng J, Chen Y, Wu T, et al. Inhibition of bleomycin-induced pulmonary fibrosis in SD rats by sea cucumber peptides. J Sci Food Agric. 2024;104:2876–87. 10.1002/jsfa.13180. 38018265 10.1002/jsfa.13180

[164] Li Q, Zhou HB, Liu JQ, Bai WF, Wang J, Yang ZJ, et al. The intervention effect of Amygdalus mongolica oil on the metabolomics and intestinal flora in pulmonary fibrosis. Front Pharm. 2022;13:1037563 10.3389/fphar.2022.1037563. 10.3389/fphar.2022.1037563 PMC9663812 36386194

[165] Wang T, Li S, Wu Y, Yan X, Zhu Y, Jiang Y, et al. Mechanistic investigation of xuebijing for treatment of paraquat-induced pulmonary fibrosis by metabolomics and network pharmacology. ACS Omega. 2021;6:19717–30. 10.1021/acsomega.1c02370. 34368559 10.1021/acsomega.1c02370 PMC8340419

